# Disentangling the neural underpinnings of response inhibition in disruptive behavior and co-occurring ADHD

**DOI:** 10.1007/s00787-025-02638-4

**Published:** 2025-01-18

**Authors:** Gülhan Saraçaydın, Daan van Rooij, Renee Kleine-Deters, Marieke Messchendorp, Jilly Naaijen, María José Penzol, Mireia Rosa, Pascal-M. Aggensteiner, Sarah Baumeister, Nathalie Holz, Tobias Banaschewski, Melanie Saam, Ulrike M. E. Schulze, Arjun Sethi, Michael Craig, Josefina Castro-Fornieles, Celso Arango, Susanne Walitza, Julia Werhahn, Daniel Brandeis, Barbara Franke, I. Hyun Ruisch, Jan K. Buitelaar, Andrea Dietrich, Pieter J. Hoekstra

**Affiliations:** 1 Department of Child and Adolescent Psychiatry, University Medical Center Groningen, University of Groningen, Groningen, The Netherlands; 2 Department of Cognitive Neuroscience, Donders Institute for Brain, Cognition and Behaviour, Radboud University Medical Center, Nijmegen, The Netherlands; 3 Centre for Cognitive Neuroimaging, Donders Institute for Brain, Cognition and Behaviour, Radboud University Medical Center, Nijmegen, The Netherlands; 4 Child and Adolescent Psychiatry Department, Hospital General Universitario Gregorio Marañón School of Medicine, Universidad Complutense, IiSGM, CIBERSAM, Madrid, Spain; 5 Department of Child and Adolescent Psychiatry and Psychology, Clínic Institute of Neurosciences, Hospital Clínic de Barcelona, IDIBAPS, Barcelona, Spain; 6 Department of Child and Adolescent Psychiatry and Psychotherapy, Central Institute of Mental Health, Medical Faculty Mannheim, Heidelberg University, Mannheim, Germany; 7 Department of Child and Adolescent Psychiatry and Psychotherapy, University of Ulm, Ulm, Germany; 8 Department of Forensic & Neurodevelopmental Sciences, Institute of Psychiatry, Psychology & Neuroscience, King’s College London, London, UK; 9 Department of Child and Adolescent Psychiatry and Psychology, Clínic Institute of Neurosciences, Hospital Clínic de Barcelona, 2017SGR881, University of Barcelona, CIBERSAM, IDIBAPS, Barcelona, Spain; 10 Department of Child and Adolescent Psychiatry and Psychotherapy, Psychiatric Hospital, University of Zurich, Zurich, Switzerland; 11 Neuroscience Center Zurich, University and ETH Zurich, Zurich, Switzerland; 12 Department of Human Genetics, Donders Institute for Brain, Cognition and Behaviour, Radboud University Medical Center, Nijmegen, The Netherlands; 13 Department of Psychiatry, Donders Institute for Brain, Cognition and Behaviour, Radboud University Medical Center, Nijmegen, The Netherlands; 14 Karakter Child and Adolescent Psychiatry University Center, Nijmegen, The Netherlands

**Keywords:** Disruptive behavior disorder, Attention-deficit/hyperactivity disorder, Dimensional approach, Response inhibition, Functional magnetic resonance imaging

## Abstract

While impaired response inhibition has been reported in attention-deficit/hyperactivity disorder (ADHD), findings in disruptive behavior disorders (DBDs) have been inconsistent, probably due to unaccounted effects of co-occurring ADHD in DBD. This study investigated the associations of behavioral and neural correlates of response inhibition with DBD and ADHD symptom severity, covarying for each other in a dimensional approach. Functional magnetic resonance imaging data were available for 35 children and adolescents with DBDs (8–18 years old, 19 males), and 31 age-matched unaffected controls (18 males) while performing a performance-adjusted stop-signal task. No significant association was found between behavioral performance and symptom severities. However, contrasting successful inhibition with failed inhibition revealed that DBD and ADHD symptom severity was associated with greater activation in the right inferior frontal regions and reduced activation in the bilateral striatal regions, respectively. During successful inhibition versus go-trials, ADHD symptom severity was associated with the left lateral occipital cortex activation. The contrast of failed inhibition versus go-trials revealed reduced activation in the right frontal and left parietal regions associated with DBD symptom severity while ADHD symptom severity was associated with bilateral precunei, dorsolateral prefrontal and left posterior parietal regions. Except for the right inferior frontal regions during successful versus failed inhibition, all clusters were also found to be inversely associated with the other dimension of interest (i.e., DBD or ADHD symptoms). Opposite direction of the associations between DBD and ADHD symptom severity, and fronto-parietal and fronto-striatal activation suggest unique contributions of DBD and ADHD to the neural correlates of response inhibition.

**Supplementary Information:**

The online version contains supplementary material available at 10.1007/s00787-025-02638-4.

## Introduction

Conduct disorder (CD) and oppositional defiant disorder (ODD) are two related types of disruptive behavior disorders (DBDs) and are among the most frequently diagnosed disorders in childhood and adolescence, with a prevalence ranging from 1 to 11% [1, 2]. ODD is defined by a recurrent pattern of negativistic, defiant, disobedient, and hostile behavior directed at authority figures, whereas CD is a more severe disorder characterized by a repetitive and persistent pattern of behavior that involves the violation of fundamental rights of others, social norms, or laws [2]. Attention-deficit/hyperactivity disorder (ADHD) is one of the common comorbidities in DBDs, with reported comorbidity rates ranging up to 30% [3, 4], and is characterized by a persistent pattern of inattention and/or hyperactivity-impulsivity that interferes with daily functioning [2].

Impairments in executive functioning are important features of ADHD and DBDs, referring to deficits in self-regulatory and higher-order cognitive processes that are required for the planning and execution of purposeful and self-serving actions [5]. Response inhibition, one of the core components of executive functioning [5], is defined as the ability to stop or postpone a dominant, automatic, or prepotent response [6]. While impaired response inhibition has often been regarded as a primary deficit specific to ADHD [7–11], the relationship between response inhibition and DBDs is less well established. Findings from behavioral and neuroimaging studies have been inconsistent, with one of the key issues remaining whether impaired response inhibition in DBDs is independent of comorbid ADHD [7, 8].

The stop-signal task has traditionally been used to probe the mechanisms of response inhibition that occur when a motor response, considered inappropriate in a given context, must be inhibited [9]. In an early meta-analysis of behavioral studies, slower inhibitory control, implying impaired response inhibition during the stop-signal task, was reported not only in children with ADHD but also in children with CD, irrespective of comorbid ADHD [10]. However, later studies found deficits in response inhibition specifically associated with ADHD symptoms or more pronounced in individuals with ADHD relative to those with DBDs, regardless of ADHD comorbidity [11–14]. On the other hand, decreased inhibitory control has also been shown in more recent studies of adolescents with DBDs, independent of comorbid ADHD [15, 16]. Notably, another study even found that adolescents with both ADHD and ODD showed poorer inhibitory control during the stop-signal task than those with ADHD, while no significant difference between adolescents with ADHD-only and healthy controls was observed [17]. However, the majority of stop-signal studies including the aforementioned ones did not incorporate assessment of neuroimaging activation during response inhibition performance.

Previous neuroimaging studies in healthy subjects have identified a large widespread network encompassing prefrontal and striatal regions which were hypothesized to be involved in the implementation of inhibitory control, and parietal areas which have been proposed to play a prominent role in the modulation of attentional processing during successful inhibition [18–20]. To date, only few functional magnetic resonance imaging (fMRI) studies have investigated response inhibition during the stop-signal task in children and adolescents with DBDs [8, 18–20]. *Increased* activation in the frontal and subcortical areas including the inferior frontal gyrus, posterior cingulate cortex, and striatum, was reported in subjects with CD compared to healthy controls during successful inhibition [18]. In another study, CD symptoms were found to be associated with poor response inhibition and *decreased* activation in bilateral frontal regions during failed inhibition [12]. Children with ODD showed *reduced* activation in the inferior and middle frontal gyri but *hyperactivation* in the dorsolateral prefrontal cortex [20]. Both hyperactivation and hypoactivation can be considered as a sign of atypical neural functioning as hypoactivation might be related to its functional incapability whilst hyperactivation might be interpreted as inefficient functioning [21]. Thus, those studies point to aberrant activity of the brain regions mainly involved in response inhibition network in DBD population. However, they implemented different experimental paradigms to assess response inhibition, and none of them had taken ADHD comorbidity into account.

In contrast to the abovementioned neuroimaging findings in relation to DBDs, several meta-analyses on neural correlates of executive functioning in children and adolescents with ADHD during stop-signal tasks have consistently demonstrated *reduced* activation in two spatially distributed networks: fronto-parietal and fronto-striatal [22–28]. The former encompasses the prefrontal cortex, anterior cingulate cortex, and superior temporal and parietal regions, while the latter includes the prefrontal cortex, pre-supplementary motor area, striatum, and subthalamic nucleus. *Increased* activity was found in the right inferior parietal and superior temporal regions, suggesting the involvement of compensatory mechanisms to overcome response inhibition-related impairments [18, 27, 29, 30].

CD and ODD can be seen as developmentally related disorders that form a severity continuum of DBD-related behaviors [31]. Similarly, ADHD symptoms may be considered as continuous traits in the general population, with ODD, CD and ADHD as the most severe conditions at the extreme end of the spectrum [32, 33]. In the past decade, there has been an increasing use of a dimensional approach as opposed to using diagnostic categories to investigate the relationship between different conditions, their interactions, and co-occurrence. The dimensional approach explains comorbidity by the fact that a person has strong manifestations of symptoms on different dimensions rather than having two or more disorders [34, 35]. To capture the full range of symptomology, it could be more informative to investigate the possible effects of DBD and ADHD symptoms on inhibitory control through a dimensional perspective [36, 37].

No studies to date have attempted to disentangle the relation of coexisting DBD and ADHD symptoms with neural activation related to response inhibition. In this study, we aimed to fill this gap in the literature by dimensionally investigating DBD (conduct and oppositional defiant symptoms) and ADHD (inattention and hyperactivity-impulsivity symptoms) in relation to the behavioral and neural correlates of response inhibition. We used a performance-adjusted stop-signal task in a sample consisting of children and adolescents with DBDs (age 8–18 years) and age-matched unaffected controls. Considering behavioral performance, we expected that DBD and ADHD dimensions would be related to decreased inhibitory control. Based on previous fMRI studies in children and adolescents with DBDs, ADHD, or the broader spectrum of externalizing problems [18–30], we hypothesized that the DBD- and ADHD-related dimensions would be associated with aberrant (either hypo or hyper) neural activation in the cortical, namely prefrontal and parietal regions, and subcortical, namely striatal areas, involved in the response inhibition network.

## Method

### Participants

Participants with DBDs and unaffected controls aged 8–18 years were recruited across four sites [Nijmegen (Radboud University Medical Center and the Donders institute for Brain, Cognition and Behavior, Nijmegen, The Netherlands), Mannheim (Central Institute of Mental Health, Mannheim, Germany), London (Centre for Neuroimaging Sciences, Institute of Psychiatry, Psychology and Neuroscience, King’s College London, London, United Kingdom; Department of Child Psychiatry, Institute of Psychiatry, Psychology and Neuroscience, King’s College London, London, United Kingdom), and Barcelona (Department of Child and Adolescent Psychiatry and Psychology, Neurosciences Institute, Hospital Clinic de Barcelona, Barcelona, Spain)], as part of the EU FP7 MATRICS and Aggressotype projects (http://www.matrics-project.eu; http://www.aggressotype.eu/). Detailed information about recruiting sites and subjects included in the analysis can be found in supplementary data (Table SI1).

Participants were included as DBD cases if they met criteria for an ODD and/or CD diagnosis on the Kiddie Schedule for Affective Disorders and Schizophrenia Present and Lifetime Version (K-SADS-PL) [38], based on the Diagnostic and Statistical Manual of Mental Disorders (fifth edition, DSM-5) criteria, or scored higher than a clinical cut-off (T ≥ 70) on the aggression or rule-breaking subscale of the Teacher Report Form (TRF), Youth Self Report (YSR), or Child Behavior Checklist (CBCL) [39]. Inclusion criteria for unaffected controls were the absence of a DSM-5 Axis-I disorder based on the K-SADS-PL [38], and aggression scores T < 70 on the aggression or rule-breaking subscales of the TRF, YSR, and CBCL [39]. IQ of the participants was estimated by using four subtests (vocabulary, similarities, block design, and picture completion/matrix reasoning) of the Wechsler Intelligence Scale for Children III or IV [40, 41].

Exclusion criteria for both unaffected controls and DBD cases included contraindications for MRI, an IQ < 70 [40, 41], and/or a primary DSM-5 diagnosis of psychosis, bipolar disorder, major depression, or anxiety disorder. After the description of the study, written informed consent was obtained from parents, and from participants who were older than 12 years old, in accordance with local legislation. The study has been approved by the local ethics committee at each site. Information about medication use was collected during the interview with the parents on the testing day.

### Rating scales

The CBCL is a widely used 113-item parent-completed questionnaire evaluating behavioral competency and behavioral problems in children within the past six months [39]. Responses are rated on a three-point Likert scale. In this study, DBD symptom severity score was calculated as the sum of all eight items for conduct problems and all five items for oppositional defiant problems. The Swanson, Nolan, and Pelham Rating Scale (SNAP-IV) is a 26-item parent-rated measure used to assess ADHD and ODD symptoms in children [42]. It includes nine items for the symptoms of ADHD hyperactive-impulsive type and nine items for the symptoms of ADHD inattentive type as specified in DSM-V. The items are scored on a four-point Likert scale. ADHD symptom severity score was calculated as the sum of average rating-per-item subscale scores for the inattention and hyperactivity-impulsivity.

To examine possible confounding effects of aggressive behaviors as well as callousness, uncaringness, and unemotionality, the self-reported the Reactive-Proactive Aggression Questionnaire (RPQ) [43], and the Inventory of Callous-Unemotional Traits (ICU) [44] were used to measure reactive and proactive aggression, and callous-unemotional traits, respectively.

### fMRI paradigm: stop-signal task

The stop-signal task has been used for many years to probe the behavioral and neural mechanisms of response inhibition [9]. Using the visual version of this paradigm, participants performed a two-choice reaction task in which they were required to inhibit their ongoing response when they were presented an additional stimulus, a stop-signal, following a target stimulus, a go-signal. In trials with a go-signal (go trials), a horizontal left- or right-pointing arrow was displayed for a maximum of 2500 ms or until a button was pressed. Depending on the direction of the arrow, participants were asked to respond as quickly as possible by pressing the left or right button using their right index finger. In 20–25% of trials (stop trials), an arrow pointing upwards was presented after a variable delay after the appearance of the go-signal, the so-called “stop-signal delay”, indicating that participants were required to interrupt the already initiated reaction and to suppress their responses. We used a performance-adjusted stop-signal task in which the initial 250-ms stop-signal delay was continuously adapted to the individual response time of the participant by increasing or decreasing it 50 ms after a successful or unsuccessful stop trial, respectively, so that an approximately 50% success rate on the stop trials for all participants was achieved [45]. Before scanning, subjects were asked to complete a short practice session of the stop-signal task. The experimental task consisted of 156 go- and 40 stop-trials and the duration of the experiment was approximately 12 min, with a range of 10 to 13 min.

Response inhibition performance was measured by the stop-signal reaction time (SSRT), which was calculated by subtracting the mean stop-signal delay from mean reaction time [45]. Other task outcomes of interest were mean reaction time to go-stimuli (MRT), intra-individual coefficient of variation of reaction time to go stimuli (ICV), the percentage of omission errors in the go trials (Go error), and the percentage of commission errors in the stop trials (Stop error).

### MRI acquisition and processing

MRI scans were performed using 3-Tesla MR scanners across imaging sites with the scanning parameters reported in supplementary Table SI2 and Table SI3. The fMRI data were preprocessed using FSL FEAT (FMRIB’s Software Library, www.fmrib.ox.ac.uk/fsl; fMRI Expert Analysis Tool, version 6.0) [46]. Preprocessing included removal of the first five volumes of each acquisition, motion correction to the middle volume, slice-timing correction, and spatial smoothing using a 6-mm Gaussian kernel. After preprocessing, additional motion artifacts were removed by applying the ICA-AROMA protocol, which is freely available through GitHub (https://github.com/maartenmennes/ICA-AROMA) [47].

### Behavioral data analysis

Multiple linear regression analyses were conducted to examine the relationship between behavioral outcomes of the stop-signal task ((i) mean reaction time to go-stimuli, (ii) intra-individual coefficient of variation of reaction time to go stimuli, (iii) percentage of omission errors, iv) stop-signal reaction time, v) percentage of stop errors) with the DBD and ADHD symptom severity scores while controlling for sex, age, IQ, scanning site, and the other respective score dimension, using the lme4 package in R (version 4.0.2) [48, 49]. In total, 10 analyses (5 stop-signal task behavioral outcomes in relation to 2 symptom scores) were performed.

### fMRI data analysis

The individual-level analysis was conducted across all participants using a general linear model in FSL FEAT [46]. The model included three regressors of interest (onsets of go-signals in correct go-trials successful and unsuccessful stop trials, separately) and two regressors of no interest (onsets of go-signals in incorrect and miss go trials together, and white matter and cerebrospinal fluid time series as nuisance regressors). The motion parameters were not included as regressors of no interest as we excluded participants with excessive head motion (> 3 mm) and applied ICA-AROMA for the automatic detection and removal of motion-related artifacts from the fMRI data [47]. Each regressor was convolved with a canonical hemodynamic response function, and the temporal derivatives of the regressors were also added as regressors in the model. Activation maps of three complementary contrasts of interest were calculated and spatially normalized to the MNI template to be used in group-level analysis. The three contrasts were: successful inhibition versus successful go (successful stop-trials versus successful go-trials to isolate the activation of successful inhibition of motor responses versus processing of a stimulus (the go-trial serves as reference), to identify brain regions that are activated in successful response inhibition), failed inhibition versus successful go (unsuccessful stop-trials versus successful go-trials to isolate activation of failed inhibition of motor responses versus processing of a stimulus (again, the go-trial serves as reference), to identify brain regions that are activated when the participant fails to inhibit a prepotent response), and successful inhibition versus failed inhibition (successful stop-trials versus unsuccessful stop-trials to isolate activation differences between successful and failed inhibition of motor responses, to identify brain regions where activation is different in successful and failed response inhibition).

In the group-level analysis, mixed-effects analysis using FLAME 1 (FSL’s Local Analysis of Mixed Effects) [50] were conducted to generate t-contrasts with the contrasts mentioned above to determine the influence of DBD and ADHD symptom severity on response inhibition-related brain activation during the stop-signal task. For this, we used either the DBD *or* the ADHD symptom severity scores as regressor of interest, while controlling for sex, age, IQ, scanning site and the other (DBD *or* ADHD) symptom dimension. A total of 6 analyses were performed (3 fMRI contrasts for 2 predictors of interest). To investigate global, cluster-wise effects across the brain, a cluster-forming threshold was applied to Z-statistic images at the FSL default value of Z = 2.3. To correct for multiple comparisons across the whole brain, family-wise error (FWE) corrected P values were computed for each cluster and thresholded at *P* = 0.05. Then, for every participant, the mean parameter estimates for all clusters positively or negatively associated with DBD or ADHD symptom severity were extracted, and post-hoc analyses were carried out using R software to explore the associations further [48]. In the case of a cluster associated with ADHD symptom severity scores, the link between cluster means and DBD symptom severity scores was also explored, and vice versa. This was done to investigate, whether clusters of brain activity identified to be relevant for one disorder (even while controlling for the other disorders’ symptomatology), could be potentially also relevant for the other disorder. For these post-hoc analyses, False Discovery Rate (FDR) correction was applied (FDR P-value threshold 0.05) to the P-values of the regression beta coefficients for the symptom scores as predictor in relation to the brain cluster means as outcome.

### Correlation of behavioral performance with brain activation

In order to establish a link between neural activity and behavioral performance, partial correlations between parameter estimates extracted from the clusters and behavioral measures ((i) mean reaction time to go-stimuli, (ii) intra-individual coefficient of variation of reaction time to go stimuli, (iii) percentage of omission errors, iv) stop-signal reaction time, v) percentage of stop errors) were calculated, partialling-out the effect of sex, age, IQ, scanning site, and DBD and ADHD symptom severity scores. A total of 45 correlations were computed (9 brain clusters with 5 behavioral measures).

### Analyses of potential confounding factors and sensitivity analyses

To rule out a possible confounding effect of aggressive behavior and callous-unemotional traits among the participants, additional sensitivity analyses for the identified brain clusters were conducted by additionally correcting for total scores on the RPQ [43] or the ICU [44], in addition to sex, age, IQ, and scanning site, and DBD and ADHD symptom severity as the variables of interest (see Supplement I, available online). A total of 36 analyses (9 brain clusters with 2 symptom scores with additional correction for RPQ scores and 9 brain clusters with 2 symptom scores with additional correction for ICU scores) was performed here.

To exclude a potential effect of stimulant (methylphenidate and/or lisdexamphetamine) use, identified brain clusters were analyzed after excluding participants who used stimulant medication on the testing day (see Supplement I, available online). A total of 18 analyses (9 brain clusters with 2 symptom scores) was performed here.

To investigate whether the identified relations between task-related brain activation and DBD and/or ADHD symptom severity were driven by residual motion artefacts in the data, we computed the average FD-value for the participants included in the analyses and analyzed the correlation of the FD-values with ADHD and DBD symptom severity.

Furthermore, differences between in- and excluded participants on a number of key variables were investigated because of the relatively large number of exclusion due to data quality control.

## Results

### Subject characteristics

From the 103 participants (*n* = 43 unaffected controls and *n* = 60 DBD) with available data, 37 had to be excluded because of excessive head movement (> 3 mm) in the scanner (*n* = 30 [29%]; *n* = 10 [23%] unaffected controls and *n* = 20 [33%] DBD), and extreme scores deviating more than 2 times the standard deviation of the grand mean on one or more of the task variables (i.e., MRT, ICV, and SSRT; *n* = 7 [7%]; *n* = 2 [5%] unaffected controls and *n* = 5 [8%] DBD). As a result, a total of 66 participants (*n* = 31 unaffected controls and *n* = 35 DBD) were left for analysis. The excluded participants were not significantly different from those included in the analyses in terms of sex, age, IQ, and DBD and ADHD symptom severity. Sample characteristics are presented in Table 1. Basic demographic information of the participants from the contributing sites can be found in Table SI3 (available online).

### Task performance

Neither DBD nor ADHD symptom severity were associated with any behavioral outcome, including the stop-signal reaction time (SSRT). Nevertheless, as can be seen from Table 1, the participants with DBD showed higher intra-individual variation of reaction time to Go-stimuli (ICV).

**Table 1:** Demographic characteristics and stop-signal task outcome measures

	**All (*N*** **=** **66)**				**Unaffected controls (*N*** **=** **31)**		**DBD (*N*** **=** **35)**^**e**^		**Test statistics**	***p*** **-value**
*Demographic characteristics*										
Sex (female/male)	22 (33%)/44 (67%)				13 (42%)/18 (58%)		9 (26%)/19 (74%)		X^2^ = 0.603	0.437
Stimulant use (yes/no) ^a^	12 (18%)/54 (82%)				0/31 (100%)		12 (34%)/23 (66%)			
Handedness (right/left)	58 (88%)/8 (12%)				26 (84%)/5 (16%)		32 (91%)/3 (9%)		X^2^ = 0.882	0.348
	**Mean**	**SD**	**Min**	**Max**	**Mean**	**SD**	**Mean**	**SD**		
Age in years	13.5	2.51	8.31	18.35	13.52	2.23	13.48	2.77	t(64)=-0.056	0.956
IQ ^b^	104.59	12.57	75.05	133.27	109.3	12.15	100.43	11.57	t(64)=-3.037	0.004
DBD score ^c,^ *	11.38	9.27	0	32	3.48	4.61	18.38	6.2	t(64) = 10.952	< 0.001
ADHD score ^d,^ *	18.41	13.82	0	49	8.16	8.48	27.49	10.99	t(64) = 7.926	< 0.001
*Stop-signal task outcomes*										
MRT *(ms)*	503.7	107.29	340.2	854.9	481	73.64	523.8	127.81	β = 0.324	0.152
ICV *(ms)*	0.27	0.06	0.16	0.45	0.25	0.06	0.29	0.06	β = 0.616	0.001
Go error (%)	5.92	4.64	0	18.59	3.52	3.63	6.17	5.1	β = 0.365	0.096
SSRT *(ms)*	223.4	125.69	2.79	498.99	251.01	111.45	198.94	133.91	β=-0.211	0.311
Stop error (%) ^f^	53.03	7.17	40	80	53.63	7.77	52.5	6.67	β=-0.372	0.079

DBD, participants diagnosed with disruptive behavior disorder; MRT, mean reaction time on successful go trials; ICV, intra-individual coefficient of variation of reaction time to go stimuli; Go error, omission error percentage on go trials; SSRT, stop-signal reaction time; Stop error, error percentage on stop trials; X^2^, chi-square; t, independent-samples t-test; β, standardized regression coefficient

^a^ Methylphenidate and/or lisdexamphetamine use. Seven children with DBD used antidepressants

^b^ Based on the Wechsler Intelligence Scale for Children III or IV [40, 41]

^c^ Sum of the scores on the conduct and oppositional defiant problems subscales of the Child Behavior Checklist (CBCL) [39]

^d^ Sum of the scores on the inattention and hyperactivity-impulsivity subscales of the Swanson, Nolan, and Pelham Rating Scale [42]

^e^ The DBD group included 14 children with oppositional-defiant disorder (ODD; 4 of them with co-occuring CD and/or ADHD), 8 children with conduct disorder (CD; 6 of them with co-occuring ODD and/or ADHD) and 10 children with attention-deficit hyperactivity disorder (ADHD; 7 of them with co-occuring ODD and/or CD, 3 of them classified as DBD-case based on their Teacher Report Form (TRF), Youth Self Report (YSR) or Child Behavior Checklist (CBCL) score);10 children with co-occurring attention-deficit hyperactivity disorder (ADHD) and 8 with conduct disorder (CD): oppositional-defiant disorder only (ODD) *N* = 10, ODD + ADHD *N* = 4, ODD + CD *N* = 3, ODD + CD + ADHD *N* = 3, and CD *N* = 2 As explained in **Methods** (Participants), DBD cases were defined based on ODD/CD/ADHD diagnoses according to the Kiddie Schedule for Affective Disorders and Schizophrenia Present and Lifetime Version [38], and when children scored above the clinical cut-off (T ≥ 70) on the aggression or rule-breaking subscale of the Teacher Report Form (TRF), Youth Self Report (YSR), or CBCL [39] *N* = 13, of whom 3 had co-occurring ADHD

^f^ The stop error rate was designed to be around 50% for all participants by adjusting difficulty of the stop-signal task based on performance

* DBD and ADHD scores were positively correlated, r(64) = 0.79, *p* < 0.001

### Brain activation

#### Successful inhibition versus successful go trials

**Fig. 1:**
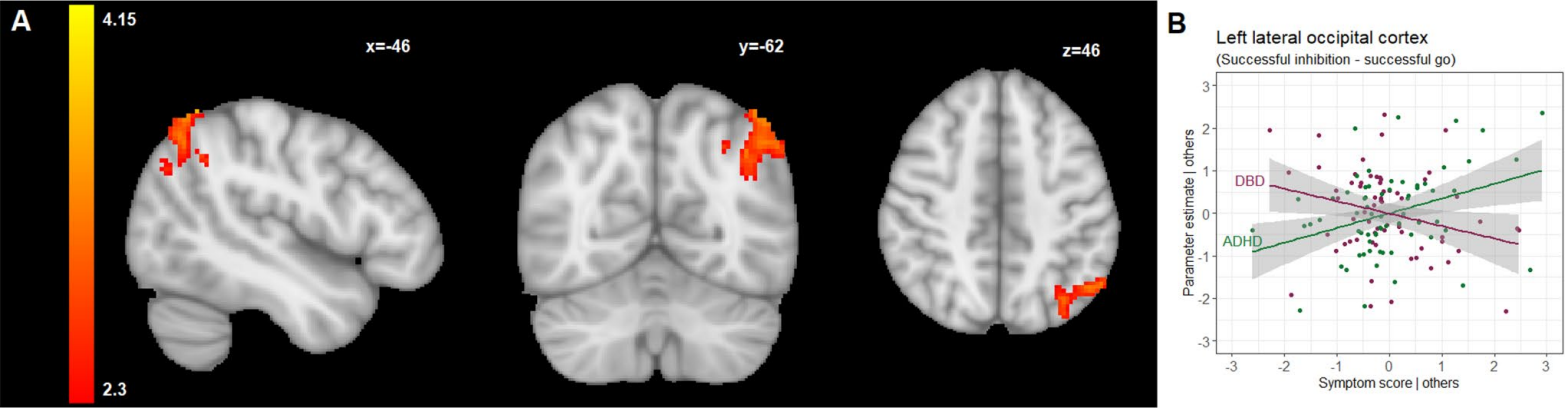
(**A**) Brain regions that were positively correlated with ADHD symptom severity scores during successful inhibition versus successful go trials, shown in radiologic view with the right brain shown on the left; (**B**) A partial regression plot for the multiple linear regression model reflecting the partial correlation coefficient between parameter estimates extracted from the cluster shown in (**A**), and symptom severity scores after adjustment for sex, age, IQ, scanning site, and the other respective score dimension. Points represent the partial residuals, the solid line indicates predicted values from the model, and the gray area indicates 95% confidence interval of the predicted values. *For comprehensiveness*,* we also showed the relation between the cluster parameter estimates and the other disorder (so in case of a DBD-associated cluster*,* we also showed the link with ADHD scores and vice versa)*,* therefore each plot shows 2 line graphs.* See also Table 2 for an overview of the clusters identified in this contrast

Higher ADHD symptom severity was associated with increased activation in the left superior lateral occipital cortex (β = 0.564, false discovery rate corrected-*p* = 0.007) (Table 2; Fig. 1 and supplementary Figures SI1-SI3). Post-hoc analysis of the parameter estimates extracted from this cluster further showed a negative association between the cluster activity and DBD symptom severity (β=-0.427, false discovery rate corrected-*p* = 0.038) (Table 2; Fig. 1).

**Table 2:** Location, maximum z-value, size and p-value of the clusters correlating with DBD and ADHD scores

Association	Brain regions	x, y, z (MNI)	Max z-value	Cluster size	Cluster p-value	Post-hoc analyses
*Successful inhibition versus successful go trials*						
ADHD score^b^ (+)	Left superior LOC	-46, -56, 60	3.63	538	0.019	DBD score: β=-0.427, p^a^=0.038 ADHD score: β = 0.564, p^a^=0.007
*Failed inhibition versus successful go trials*						
DBD score^c^ (-)	Right MFG, SFG	50, 22, 52	4.09	700	0.004	DBD score: β=-0.769, p^a^<0.001 ADHD score: β = 0.621, p^a^=0.001
DBD score^c^ (-)	Left SPL, posterior SMG, ANG, PCC	-26, -52, 36	3.78	523	0.023	DBD score: β=-0.82, p^a^<0.001 ADHD score: β = 0.611, p^a^=0.002
ADHD score^b^ (+)	Bilateral PCUN	-2, -38, 48	3.96	789	0.002	DBD score: β=-0.505, p^a^=0.007 ADHD score: β = 0.743, p^a<^0.001
ADHD score^b^ (+)	Left SPL, superior LOC	-34, -80, 50	4.15	752	0.002	DBD score: β=-0.545, p^a^=0.007 ADHD score: β = 0.575, p^a^=0.007
ADHD score^b^ (+)	Right MFG, SFG	16, 20, 44	4.07	680	0.005	DBD score: β=-0.7, p^a^<0.001 ADHD score: β = 0.776, p^a^<0.001
ADHD score^b^ (+)	Left SFG, MFG	-22, 28, 50	3.62	620	0.009	DBD score: β=-0.619, p^a^=0.003 ADHD score: β = 0.708, p^a^=0.001
*Successful inhibition versus failed inhibition*						
DBD score^c^ (+)	Right IFGtri, FPo, FO, anterior INS	50, 24, 14	3.84	945	9*10^− 4^	DBD score: β = 0.724, p^a^<0.001 ADHD score: β=-0.24, p^a^=0.197
ADHD score^b^ (-)	Bilateral THA	18, -2, 4	3.68	546	0.023	DBD score: β = 0.583, p^a^=0.003 ADHD score: β=-0.865, p^a^<0.001

MNI, Montreal Neurological Institute; DBD score, a composite DBD score; ADHD score, a composite ADHD score; (+), positive association; (-), negative association; β, standardized regression coefficient

Brain regions: ANG, angular gyrus; FO, frontal operculum; FPo, frontal pole; IFGtri, inferior frontal gyrus, triangular part; INS, insula; LOC, lateral occipital cortex; MFG, middle frontal gyrus; PCC, posterior cingulate gyrus; PCUN, precuneus; SFG, superior frontal gyrus; SMG, supramarginal gyrus; SPL, superior parietal lobule; THA, thalamus

^a^ False Discovery Rate corrected p-value [51]

^b^ Sum of the scores on the inattention and hyperactivity-impulsivity subscales of the Swanson, Nolan, and Pelham Rating Scale [42]

^c^ Sum of the scores on the conduct and oppositional defiant problems subscales of the Child Behavior Checklist [39]

#### Failed inhibition versus successful go trials

**Fig. 2:**
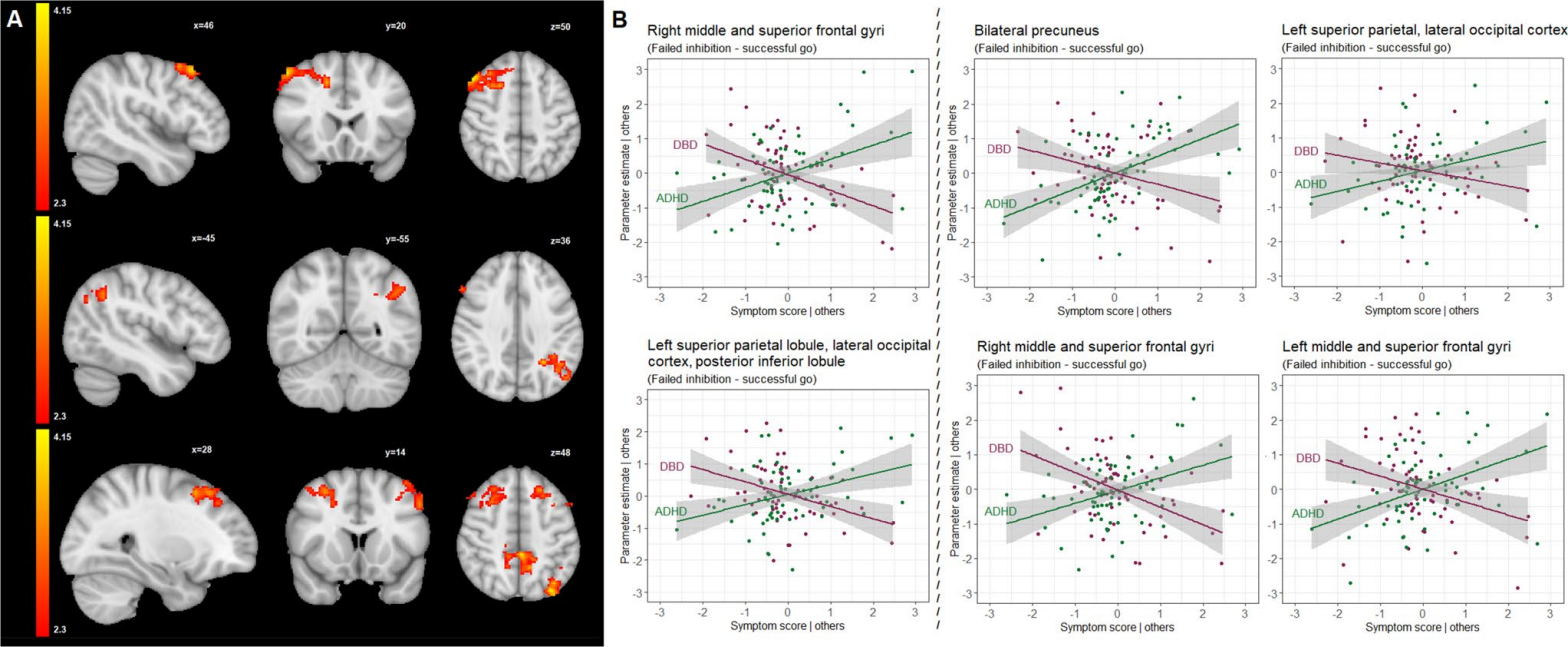
(**A**) Brain regions (two clusters) that were negatively correlated with DBD scores (upper and middle cross-sections) and brain regions (four clusters) that were positively correlated with ADHD scores (lower cross-section) during failed inhibition versus successful go trials, shown in radiologic view with the right brain shown on the left; (**B**) Partial regression plots for the multiple linear regression models reflecting the partial correlation coefficient between parameter estimates extracted from the clusters associated with DBD (left two plots) and ADHD (right four plots) symptom severity scores and shown in (**A**), and symptom severity scores after adjustment for sex, age, IQ, scanning site, and the other respective score dimension. Points represent the partial residuals, the solid lines indicate predicted values from the models, and the gray areas indicate 95% confidence interval of the predicted values. *For comprehensiveness*,* we also showed the relation between the cluster parameter estimates and the other disorder (so in case of a DBD-associated cluster*,* we also showed the link with ADHD scores and vice versa)*,* therefore each plot shows 2 line graphs*. See also Table 2 for an of the overview clusters identified in this contrast

Higher DBD symptom severity was associated with reduced activation in the left posterior superior parietal (β=-0.82, false discovery rate corrected-*p* < 0.001) and right dorsolateral prefrontal cortex (β=-0.769, false discovery rate corrected-*p* < 0.001) (Table 2; Fig. 2 and supplementary Figures SI4-SI6). Post-hoc analysis revealed that the ADHD symptom severity was positively associated with the cluster activity in the left posterior superior parietal (β = 0.611, false discovery rate corrected-*p* = 0.002) and right dorsolateral prefrontal cortex (β = 0.621, false discovery rate corrected-*p* = 0.001) (Table 2; Fig. 2).

There were four clusters that were positively associated with ADHD symptom severity. The largest cluster associated with ADHD symptom severity (β = 0.743, false discovery rate corrected-*p* < 0.001) was in the bilateral precunei where the activity was negatively correlated with DBD symptom severity (β=-0.505, false discovery rate corrected-*p* = 0.007) (Table 2; Fig. 2). The second largest cluster associated with ADHD symptom severity (β = 0.575, false discovery rate corrected-*p* = 0.007) was in the left superior parietal lobule, extending posteriorly towards lateral occipital cortex (Table 2; Fig. 2). This cluster was also negatively correlated with DBD symptom severity (β=-0.545, false discovery rate corrected-*p* = 0.007) (Table 2; Fig. 2). The other two clusters associated with ADHD symptom severity covered the right (β = 0.776, false discovery rate corrected-*p* < 0.001) and left dorsolateral prefrontal cortices (β = 0.708, false discovery rate corrected-*p* = 0.001), including the middle and superior frontal gyri (Table 2; Fig. 2 and supplementary Figures SI7-SI9). Post-hoc analyses showed that DBD scores were also negatively correlated with the activity of the right (β=-0.7, false discovery rate corrected-*p* < 0.001) and left dorsolateral prefrontal regions (β=-0.619, false discovery rate corrected-*p* = 0.003) (Table 2; Fig. 2).

#### Successful inhibition versus failed inhibition

**Fig. 3:**
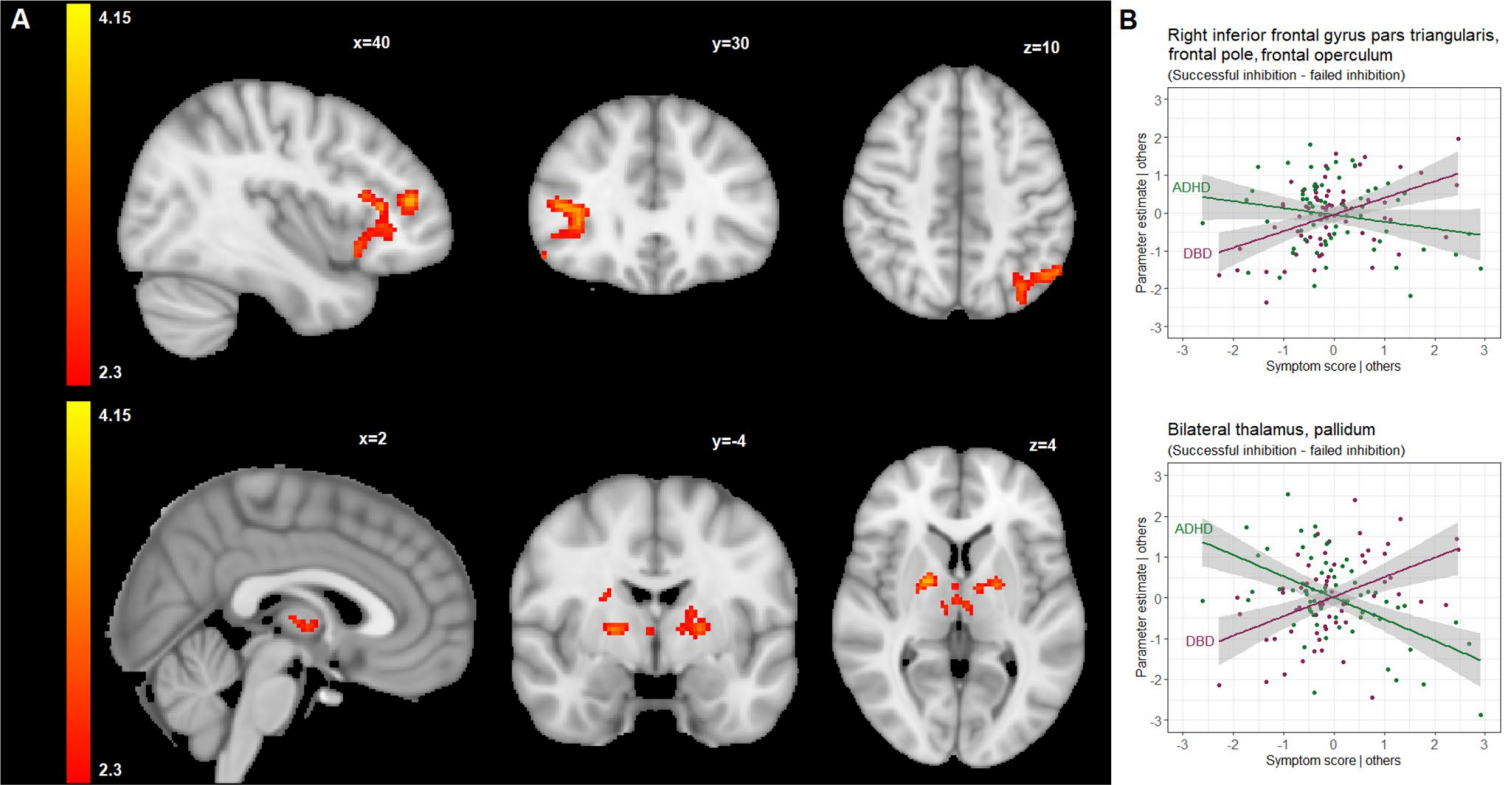
(**A**) Brain regions that were positively correlated with DBD (upper) and negatively correlated with ADHD scores (lower) during successful inhibition versus failed inhibition, shown in radiologic view with the right brain shown on the left; (**B**) Partial regression plots for the multiple linear regression models reflecting the partial correlation coefficient between parameter estimates extracted from the clusters associated with DBD (upper) and ADHD (lower) symptom severity scores and shown in (**A**), and symptom severity scores after adjustment for sex, age, IQ, scanning site, and the other respective score dimension. Points represent the partial residuals, the solid lines indicate predicted values from the models, and the gray areas indicate 95% confidence interval of the predicted values. *For comprehensiveness*,* we also showed the relation between the cluster parameter estimates and the other disorder (so in case of a DBD-associated cluster*,* we also showed the link with ADHD scores and vice versa)*,* therefore each plot shows 2 line graphs.* See also Table 2 for an overview of the clusters identified in this contrast

For the successful inhibition– failed inhibition, one cluster with a peak in the right inferior frontal gyrus, extending towards the anterior insula, was found to be positively correlated with DBD symptom severity (β = 0.724, false discovery rate corrected-p < = 0.001) (Table 2; Fig. 3 and supplementary Figures SI10-SI12).

In addition, ADHD symptom severity was negatively related to activation in the bilateral thalamus (β=-0.865, false discovery rate corrected-p < = 0.001) (Table 2; Fig. 3 and supplementary Figures SI13-SI15). Post-hoc analysis showed that the activity in this cluster was positively associated with DBD symptom severity (β = 0.583, false discovery rate corrected-*p* = 0.003) (Table 2; Fig. 3).

To summarize, across the 3 fMRI contrasts, a total of 9 brain clusters were identified: 6 in relation to ADHD symptom scores and 3 in relation to DBD symptom scores (Table 2; Figs. 1, 2 and 3).

### Association of neural activation with behavioral outcomes

Table 3 presents results for the correlations between parameter estimates extracted from clusters and behavioral measures. None of the behavioral outcomes were associated with the neural activation.

### Analyses of potential confounding factors and sensitivity analyses

After excluding participants who used stimulant medication on the testing day (Tables SI4 and SI5) and covarying for all scale scores on the RPQ and the ICU (Table SI6), the associations of the neural activation with DBD and ADHD symptom severity (except for the cluster in the left occipital cortex associated with ADHD scores) remained significant, suggesting that the main findings were in general not driven by stimulant medication and other potentially confounding variables (aggression and/or callous-unemotional traits).

The average FD-value for the participants included in the analyses was 1.152 mm (SD = 0.812). The correlation between the FD-value and ADHD symptom severity was *r*=-0.109 (*p* = 0.332) and between the FD-value and DBD symptom severity was *r*=-0.002 (*p* = 0.986). Thus, residual head motion was not correlated with ADHD/DBD symptom severity.

We did not identify significant differences between in- and excluded participants on a number of key variables, i.e., age, sex ratio, IQ, and DBD and ADHD symptom severity. Regarding task-related variables, the excluded participants had higher MRT and ICV values compared to the participants included in the analysis. Additionally, a similar pattern was observed in the number of go-errors, although this trend did not reach statistical significance. See Table SI7 for all results.

**Table 3:** Correlation analyses of neural activation with behavioral outcomes of the stop-signal task

Association	Brain regions	MRT	ICV	Go error	SSRT	Stop error
*Successful inhibition versus successful go trials*						
ADHD score^a^ (+)	Left superior LOC	-0.088	-0.017	-0.24	-0.075	0.133
*Failed inhibition versus successful go trials*						
DBD score^b^ (-)	Right MFG, SFG	-0.052	-0.133	-0.149	-0.077	0.101
DBD score^b^ (-)	Left SPL, posterior SMG, ANG, PCC	-0.091	-0.085	-0.115	-0.036	-0.065
ADHD score^a^ (+)	Bilateral PCUN	0.027	-0.046	-0.182	-0.056	0.088
ADHD score^a^ (+)	Left SPL, superior LOC	0.044	-0.064	-0.091	0.013	0.001
ADHD score^a^ (+)	Right MFG, SFG	-0.019	-0.088	-0.153	-0.059	0.156
ADHD score^a^ (+)	Left SFG, MFG	-0.141	-0.172	-0.14	-0.159	-0.039
*Successful inhibition versus failed inhibition*						
DBD score^b^ (+)	Right IFGtri, FPo, FO, anterior INS	-0.191	-0.019	-0.233	-0.109	0.091
ADHD score^a^ (-)	Bilateral THA	-0.063	-0.075	-0.155	-0.209	-0.077

The effect of sex, age, IQ, and scanning site, as well as DBD and ADHD score, respectively, were partialled-out of the correlations of ADHD score and DBD score. DBD score, a composite DBD score; ADHD score, a composite ADHD score; (+), positive association; (-), negative association; MRT, mean reaction time on successful go trials; ICV, intra-individual coefficient of variation of reaction time to go stimuli; Go error, omission error percentage on go trials; SSRT, stop-signal reaction time; Stop error, error percentage on stop trials. Reported numbers are correlation coefficients. None of these reached statistical significance

^a^ Sum of the scores on the inattention and hyperactivity-impulsivity subscales of the Swanson, Nolan, and Pelham Rating Scale [42]

^b^ Sum of the scores on the conduct and oppositional defiant subscales of the Child Behavior Checklist [39]

## Discussion

We investigated behavioral and neural correlates of response inhibition in children and adolescents with DBDs and age-matched unaffected controls, focusing on the distinct contribution of the DBD and ADHD symptom severity to the neural activity during a performance-adjusted stop-signal task. Contrary to our hypothesis on behavioral task performance, there were no significant correlations between behavioral outcomes of the task and DBD or ADHD symptom severity. Yet, consistent with our hypothesis on the brain activation patterns, the DBD and ADHD symptom dimensions were significantly associated with neural activation in different areas of fronto-parietal and fronto-striatal networks involved in response inhibition. Notably, opposite directions of the associations, observed in the same areas for DBD and ADHD symptom severity, suggest different but not necessarily unrelated mechanisms for maintaining similar cognitive performance in youth exhibiting high DBD and ADHD characteristics.

As for the behavioral performance, there was no significant difference in SSRT between DBD cases and unaffected controls. Although impaired response inhibition, as indexed by greater SSRT values, is thought to be associated with DBD and ADHD, previous stop-signal task studies not always reported shorter, but also similar SSRT values in children with DBD and ADHD compared to healthy controls [12–14, 52, 18]. These inconsistent results in the current literature might result from different samples and/or different experimental paradigms. However, consistent with previous findings, we found that DBD cases demonstrated higher variability in reaction times than unaffected controls, suggesting impaired attentional processing during the task [18].

We compared successful stop-trials to unsuccessful stop-trials to identify the neural activation associated with a successful outcome in the stop-trials. In line with our hypothesis that DBD- and ADHD-related dimensions would be associated with aberrant neural activation in fronto-striatal regions involved in response inhibition. Specifically, a cluster positively correlated with DBD symptom severity was found in the right inferior frontal regions with a main peak located in the pars triangularis of the right inferior frontal gyrus and extending towards the right anterior insula. Increased activation of the right inferior frontal gyrus, whose magnitude correlates with the efficiency of the stop response, and the right anterior insula has been repeatedly shown in many studies using the stop-signal task, supporting the notion of the right inferior frontal gyrus being crucial for stopping action and the right anterior insula contributing to inhibitory control by suppressing or slowing the response [53–57]. Interestingly, post-hoc analyses revealed that this was the only cluster associated with DBD symptoms only. Thus, the DBD dimension-related greater activation in the right inferior frontal gyrus and the neighboring regions could be associated with effortful engagement of the core nodes of the response inhibition network that enables youth with higher DBD symptoms severity, independent of ADHD symptoms, to maintain inhibitory control during the task. This contrast also revealed a subcortical cluster in the bilateral thalamus where the activity was negatively correlated with ADHD scores. Response inhibition is thought to be mediated via the indirect striatal pathway, promoting the inhibition of the thalamus activity and decreasing the cortical activity required for motor action [58, 59]. It has been shown that the thalamus showed less activation in individuals with shorter SSRT compared to those with longer SSRT [59]. Since a shorter SSRT indicates better response inhibition ability and has been previously shown to be associated with the activity of the indirect pathway, it can be speculated that the participants with high ADHD scores were more likely to deactivate the thalamus bilaterally than those with low ADHD scores to be able to inhibit their prepotent response, keeping in mind that there was no correlation between SSRT and symptom severities.

Comparing successful stop-trials with successful go-trials contrasts the brain regions engaged in a chain of processes including attention orientation, detection of the stop-signal, and inhibition of the prepotent go response. We identified a cluster within the left superior lateral occipital cortex whose activity positively correlated with ADHD symptom severity, indicating potential compensatory activation underlying the lower-order visual-perceptual processing in participants displaying more ADHD characteristics, in line with previous studies that indicated that the lateral occipital cortex is involved in attentional processes [60, 61]. During the stop-signal task, attention is directed towards a specific visual stop stimulus while inhibiting a prepotent response. Activation in the lateral occipital cortex might thus indicate the engagement of visuo-attentional mechanisms necessary for successful response inhibition.

Failed inhibition was investigated by contrasting unsuccessful stop-trials with successful-go trials. This contrast revealed that participants with higher DBD symptom severity exhibited reduced activation in the left posterior superior parietal and right dorsolateral prefrontal cortex. Superior parietal regions have been hypothesized to play an important role in inhibitory control as part of a top-down attentional control network, directing attention to task-relevant stimuli [62, 63]. Besides, the dorsolateral prefrontal cortex has long been recognized as being critical to response inhibition and attentional processing [64–66]. Accordingly, successful inhibition during the stop-signal task does not only require the ability to withhold a prepotent response but also sustained attention and proper detection and monitoring of go- and stop-signals. Attention deficits or failures in the detection and monitoring of the stop-signal, therefore, may result in inhibition failures [64]. Thus, the negative association of DBD scores with neural activation in the right dorsolateral prefrontal cortex and the left posterior parietal regions suggests a greater need for the recruitment of the regions implicated in attentional processes in youth exhibited greater DBD symptom severity.

Besides, we identified four clusters positively correlated with ADHD scores during failed inhibition. The largest cluster covers a large portion of the bilateral precunei which have been suggested to be hub of the default mode network (DMN) [67]. There is increasing evidence that the DMN, which constitutes the main active network at rest and is responsible for the modulation of attention directed to inner thoughts, would be activated abnormally in ADHD subjects when outward-directed attention is required, and thus its activity should be suppressed [68–70]. The failure of its suppression during the stop-signal task in participants with higher ADHD scores might therefore adversely affect the activity of the response inhibition networks, ultimately resulting in attention deficits and subsequent failed inhibition. The other three clusters, that were positively correlated with ADHD symptom severity, were located in the left posterior superior parietal regions with a peak in the left lateral occipital cortex extending anteriorly into the superior parietal lobule and bilateral dorsal prefrontal regions extending towards the medial surface in the right hemisphere. As discussed above, the hyperactivation of task-inactive DMN and bilateral fronto-parietal regions may suggest aberrant functioning of visuo-attentional mechanisms during failed inhibition in participants displaying greater ADHD symptomatology.

Some strengths and limitations of this study are worth mentioning regarding the interpretation of the results. A major strength was that the co-occurrence of the DBD and ADHD-related behaviors was taken into account, covarying both DBD and ADHD symptom severity for each other in a dimensional analysis, in line with the recent move towards using Research Domain Criteria (RDoC) [37]. Another strength was the robustness of the results after excluding participants who used stimulant medication on the testing day, covarying for all scale scores on the RPQ and the ICU. Further, in the fMRI analysis, orthogonalization was used to reduce multicollinearity issues in the regression model, as indicated by a high correlation between DBD and ADHD symptom severities. We thus isolated the possibly unique/independent contributions of each of them to brain activity during the stop signal task, by adjusting the effect of DBD symptom severity for ADHD symptom severity and vice versa. Although the exclusion of a relatively high number of participants from the fMRI analysis due to quality issues is a limitation, the excluded participants were not significantly different from those included in the analyses in terms of sex, age, IQ, and DBD and ADHD symptom severity. However, the resulting lowered sample size may be one explanation for the only trend-level difference between cases and controls in the number of go errors. Future multicenter studies should apply more stringent and standardized MRI data quality control procedures across the participating sites. The lack of study participants with ADHD only may also be considered as a limitation. Adding such a group to future studies would allow for investigating possible differences in neurobiological mechanisms between ADHD in the context of DBD and ADHD as such. Finally, we have considered interaction models to test for ADHD x DBD symptom severity interaction but decided to only investigate main effects because of the relatively small sample size of our study. Nevertheless, as mentioned, to isolate potentially unique effects, we have adjusted the effect of ADHD symptom severity for DBD symptom severity and vice versa.

In conclusion, using a dimensional approach, we investigated the link between co-occurring DBD and ADHD symptoms, and neural activity during a performance-adjusted stop-signal task to further elucidate the neural underpinnings of response inhibition. Our results indicated that DBD and ADHD symptom severity might be oppositely associated with aberrant neural activation in different regions of the fronto-parietal and fronto-striatal networks. As these regions were shown to be involved in both attention and inhibitory control as core nodes of the response inhibition network, distinct activation patterns associated with DBD and ADHD symptom severity may point to their unique contributions to the neural activation patterns underlying response inhibition. Therefore, our findings provide new and clinically relevant insights that should be further investigated and replicated by future research in other youth samples enriched for DBD symptoms.

## Electronic supplementary material

Below is the link to the electronic supplementary material.

Supplementary Material 1

## Data Availability

No datasets were generated or analysed during the current study.
