# Supplementary material for: Disentangling the neural underpinnings of response inhibition in disruptive behavior and co-occurring ADHD
Source: Eur Child Adolesc Psychiatry. 2025 Jan 18;34(7):2253–67. doi: 10.1007/s00787-025-02638-4 (PMC12334431; doi:10.1007/s00787-025-02638-4)
Supplement: Supplementary file 1 — Supplementary Material 1 [file 787_2025_2638_MOESM1_ESM.docx]

**Disentangling the neural underpinnings of response inhibition in disruptive behavior and co-occurring ADHD**

**Supplement 1**

**Author names and affiliations:**

Gülhan Saraçaydın, MSc ^1^, Daan van Rooij, PhD ^2, 3^, Renee Kleine-Deters, MSc ^1^, Marieke Messchendorp, MSc ^1^, Jilly Naaijen, PhD ^2, 3^, María José Penzol, MD, PhD ^4^, Mireia Rosa, PhD ^5^, Pascal-M Aggensteiner, MSc ^6^, Sarah Baumeister, PhD ^6^, Nathalie Holz, PhD ^6^, Tobias Banaschewski, MD, PhD ^6^, Melanie Saam, PhD ^7^, Ulrike M.E. Schulze, MD ^7^, Arjun Sethi, PhD ^8^, Michael Craig, PhD ^8^, Josefina Castro-Fornieles, MD, PhD ^9^, Celso Arango, MD, PhD ^4^, Susanne Walitza, MD, PhD ^10,11^, Julia Werhahn, PhD ^10,11^, Daniel Brandeis, PhD ^6, 10, 11^, Barbara Franke, PhD ^12, 13^, I. Hyun Ruisch, MD, PhD^1^, Jan K. Buitelaar, MD, PhD ^2, 14^, Andrea Dietrich, PhD ^1^ *, Pieter J. Hoekstra, MD, PhD ^1^ *

^1^ Department of Child and Adolescent Psychiatry, University Medical Center Groningen, University of Groningen, The Netherlands.

^2^ Department of Cognitive Neuroscience, Donders Institute for Brain, Cognition and Behaviour, Radboud University Medical Center, Nijmegen, The Netherlands.

^3^ Centre for Cognitive Neuroimaging, Donders Institute for Brain, Cognition and Behaviour, Radboud University Medical Center, Nijmegen, The Netherlands.

^4^ Child and Adolescent Psychiatry Department, Hospital General Universitario Gregorio Marañón School of Medicine, Universidad Complutense, IiSGM, CIBERSAM, Madrid, Spain.

^5^ Department of Child and Adolescent Psychiatry and Psychology, Clínic Institute of Neurosciences, Hospital Clínic de Barcelona, IDIBAPS. Barcelona, Spain.

^6^ Department of Child and Adolescent Psychiatry and Psychotherapy, Central Institute of Mental Health, Medical Faculty Mannheim/ Heidelberg University, Mannheim, Germany.

^7^ Department of Child and Adolescent Psychiatry and Psychotherapy, University of Ulm, Ulm, Germany.

^8^ Department of Forensic & Neurodevelopmental Sciences, Institute of Psychiatry, Psychology & Neuroscience, King’s College London, London, United Kingdom.
^9^ Department of Child and Adolescent Psychiatry and Psychology, Clínic Institute of Neurosciences, Hospital Clínic de Barcelona, 2017SGR881. University of Barcelona. CIBERSAM, IDIBAPS. Barcelona, Spain.

^10^ Department of Child and Adolescent Psychiatry and Psychotherapy, Psychiatric Hospital, University of Zurich, Zurich, Switzerland.

^11^ Neuroscience Center Zurich, University and ETH Zurich, Zurich, Switzerland.

^12^ Department of Human Genetics, Donders Institute for Brain, Cognition and Behaviour, Radboud University Medical Center, Nijmegen, The Netherlands.

^13^ Department of Psychiatry, Donders Institute for Brain, Cognition and Behaviour, Radboud University Medical Center, Nijmegen, The Netherlands.

^14^ Karakter Child and Adolescent Psychiatry University Center, Nijmegen, The Netherlands.

Correspondence to I. Hyun Ruisch, M.D, Ph.D., University of Groningen, University Medical Center Groningen, Department of Child and Adolescent Psychiatry, Lübeckweg 2, 9723 HE Groningen, The Netherlands; e-mail: i.h.ruisch@umcg.nl

*Shared senior authorship

**1. METHOD**

**1.1. Details on sites where participants were recruited**

The participants with DBDs (n=64) and unaffected controls (n=45) aged 8-18 years were recruited across four sites [Nijmegen (Radboud University Medical Center and the Donders institute for Brain, Cognition and Behavior, Nijmegen, The Netherlands), Mannheim (Central Institute of Mental Health, Mannheim, Germany), London (Centre for Neuroimaging Sciences, Institute of Psychiatry, Psychology and Neuroscience, King’s College London, London, United Kingdom; Department of Child Psychiatry, Institute of Psychiatry, Psychology and Neuroscience, King’s College London, London, United Kingdom), Barcelona (Department of Child and Adolescent Psychiatry and Psychology, Neurosciences Institute, Hospital Clinic de Barcelona, Barcelona, Spain)], as part of the EU FP7 MATRICS and Aggressotype projects (http://www.matrics-project.eu; http://www.aggressotype.eu/). Since the subjects from Nijmegen (n=2 [5%]; n=2 [9%] unaffected controls), London (n=3 [14%]; n=3 [20%] DBDs), and Barcelona (n=1 [5%]; n=1 [7%] DBDs) were excluded from this study due to absent or corrupt functional magnetic resonance imaging (fMRI) data and/or behavioral log associated with the stop-signal task, only subjects with available fMRI and behavioral data were included and presented (Table SI1).

**1.2. Cognitive screening.**

The Child Behavior Checklist (CBCL), Teacher’s Report Form (TRF), Youth Self-Report (YSR), and Swanson, Nolan, and Pelham Rating Scale (SNAP-IV) are widespread, cost- and time-efficient measuring instruments for a number of psychological disorders in childhood and adolescence [1, 2]. The CBCL was completed by parents to rate behavioral, emotional, somatic complaints and social skills of the participants. The TRF was filled out by teachers to evaluate the participants’ academic performance and adaptive functioning within a learning environment [1]. The participants 11 years of age and older used the YSR to describe their own behavioral and emotional problems [1]. The SNAP-IV was completed by parents to assess ADHD and ODD symptoms in the participants [2]. In this study, DBD symptom severity score was calculated as the sum of all eight items for conduct problems and all five items for oppositional defiant problems in the CBCL [1], whereas ADHD symptom severity score was calculated as the sum of average rating-per-item subscale scores for the inattention and hyperactivity-impulsivity in the SNAP-IV [2]. To assess aggressive behavior, reactive and proactive aggression was measured by the Reactive-Proactive Aggression Questionnaire (RPQ) [3]. The Inventory of Callous-Unemotional Traits (ICU) was used to record the callous-unemotional traits using three scales: callousness, uncaring and unemotional [4]. Full-scale IQ was estimated by using four subtests (vocabulary, similarities, block design and picture completion/matrix reasoning) of the Wechsler Intelligence Scale for Children III or IV [5, 6].

**1.3. MRI acquisition.**

Structural and functional MRI scans were performed using 3-Tesla MR scanners across imaging sites using the scanning parameters reported in Table SI2 and Table SI3, respectively.

**2. RESULTS**

A cluster-forming threshold of Z=2.3 (FSL default) and a cluster significance threshold of FWE-corrected P=0.05 were used for whole-brain analyses. Sagittal, coronal and axial views of clusters correlated with DBD and ADHD symptom severity during successful inhibition versus successful go-trials, failed inhibition versus successful go-trials, and successful inhibition versus failed inhibition were presented in Figure SI1-15.

**2.1. Evaluation of potential medication effects**

Among the participants, 34% (n=12) of DBD group were on stimulant medication at the time of the scan (Table 1). To assess the potential effect of stimulant (methylphenidate and/or lisdexamfetamine) use, behavioral and neuroimaging outcomes were reanalyzed after excluding participants who used stimulant medication on the testing day. To control family-wise type I error rate, the false discovery rate (FDR) correction was applied to the variables of interest [8]. The associations of cluster activity with DBD and ADHD symptom severity remained significant, suggesting that main neural findings were not driven by stimulant medications (Table SI5).

**2.2. Evaluation of potential confounds**

To rule out a plausible effect of aggression and callous-unemotional traits, additional post-hoc analyses were conducted on neural findings after covarying for total aggression score (reactive and proactive aggression) on the Reactive-Proactive Aggression Questionnaire [3], and total score (callousness, uncaring, and unemotional traits) on the Inventory of Callous-Unemotional Traits [4] separately in addition to sex, age, IQ, and scanning site covariates, and DBD and ADHD symptom severity as the variables of interest. The associations of cluster activity with DBD and ADHD symptom severity remained significant, suggesting that main neural findings were not driven by coexisting aggression or callous-unemotional traits (Table SI6).


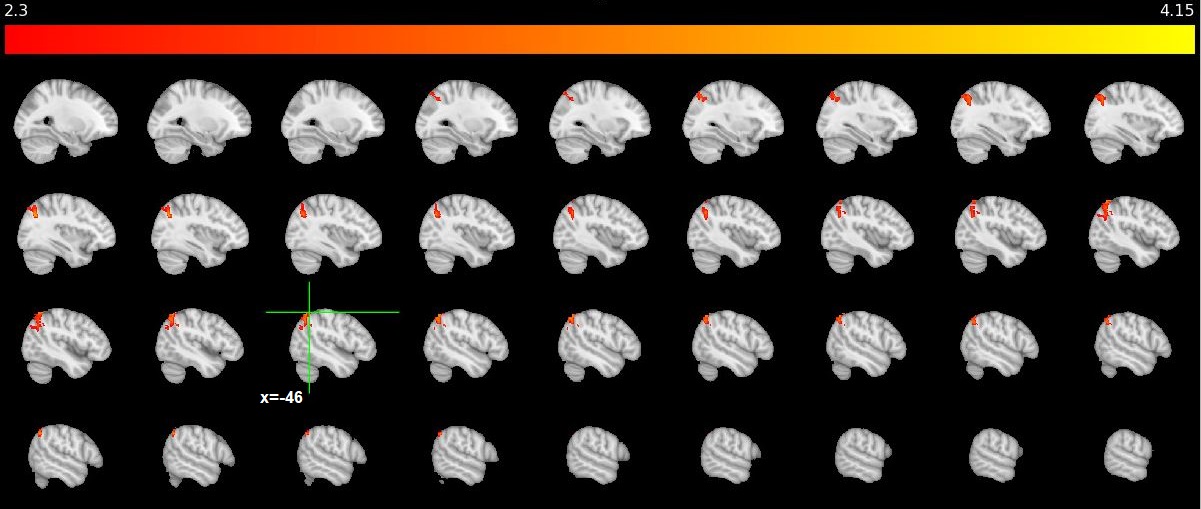


Figure SI1. Sagittal views of the cluster positively correlated with ADHD symptom severity during successful inhibition versus successful go-trials. The highlighted slice at x=-46 includes the voxel with maximum Z-value of 3.63 for that cluster.


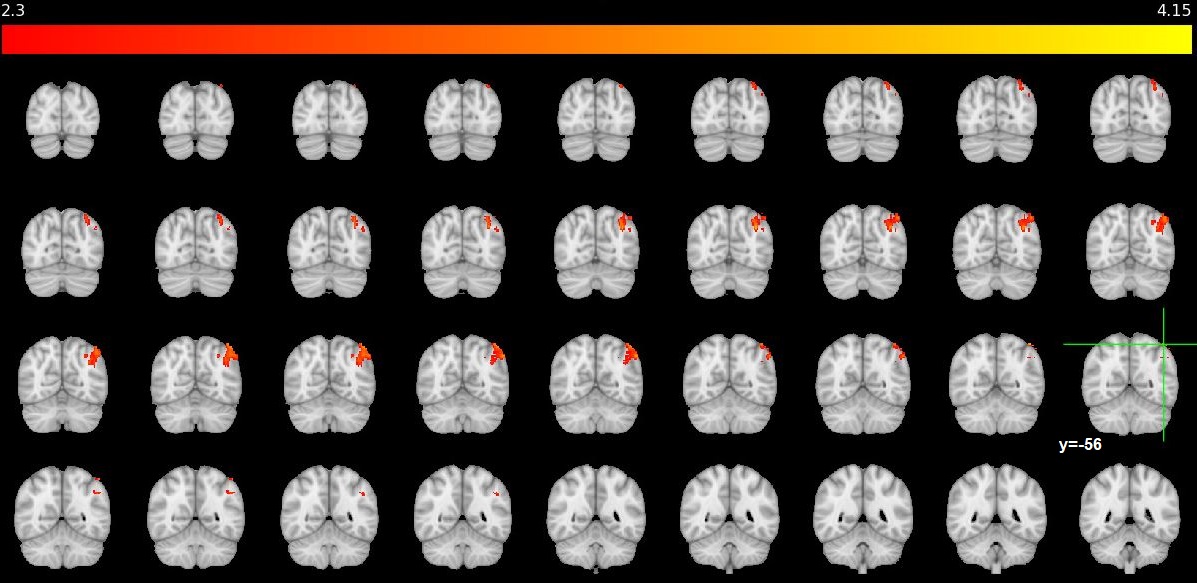


Figure SI2. Coronal views of the cluster positively correlated with ADHD symptom severity during successful inhibition versus successful go-trials, shown in radiologic view with the right brain shown on the left. The highlighted slice at y=-56 includes the voxel with maximum Z-value of 3.63 for that cluster.


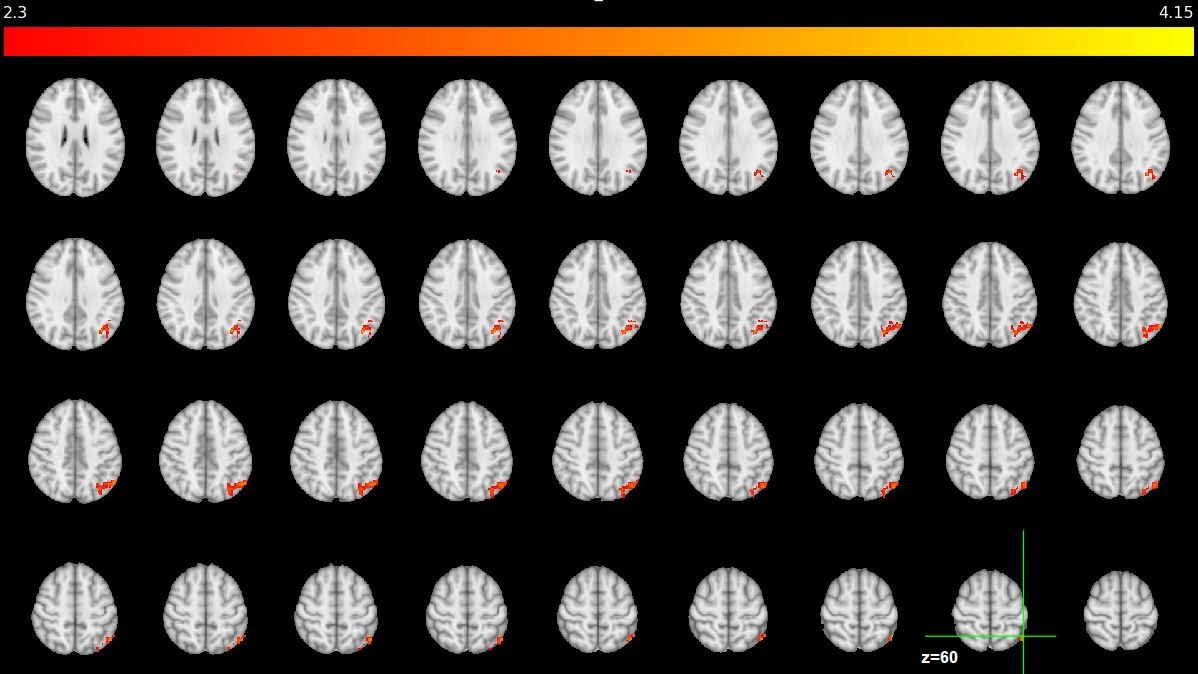


Figure SI3. Axial views of the cluster positively correlated with ADHD symptom severity during successful inhibition versus successful go-trials, shown in radiologic view with the right brain shown on the left. The highlighted slice at z=60 includes the voxel with maximum Z-value of 3.63 for that cluster.


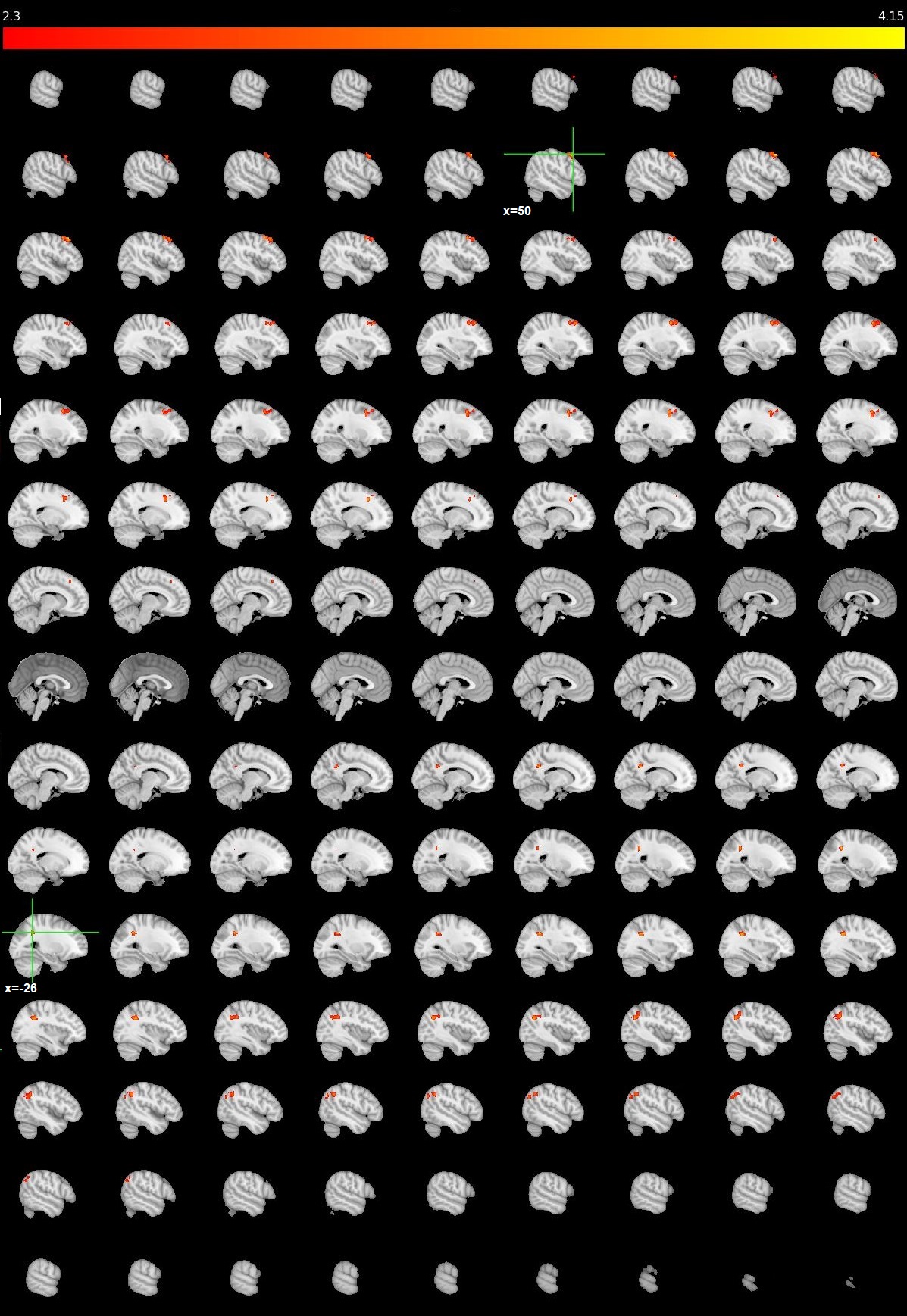


Figure SI4. Sagittal views of two clusters negatively correlated with DBD symptom severity during failed inhibition versus successful go-trials. The highlighted slices at x=50 and x=-26 include the voxel with maximum Z-value of 4.09 and 3.78, respectively, for these two clusters.


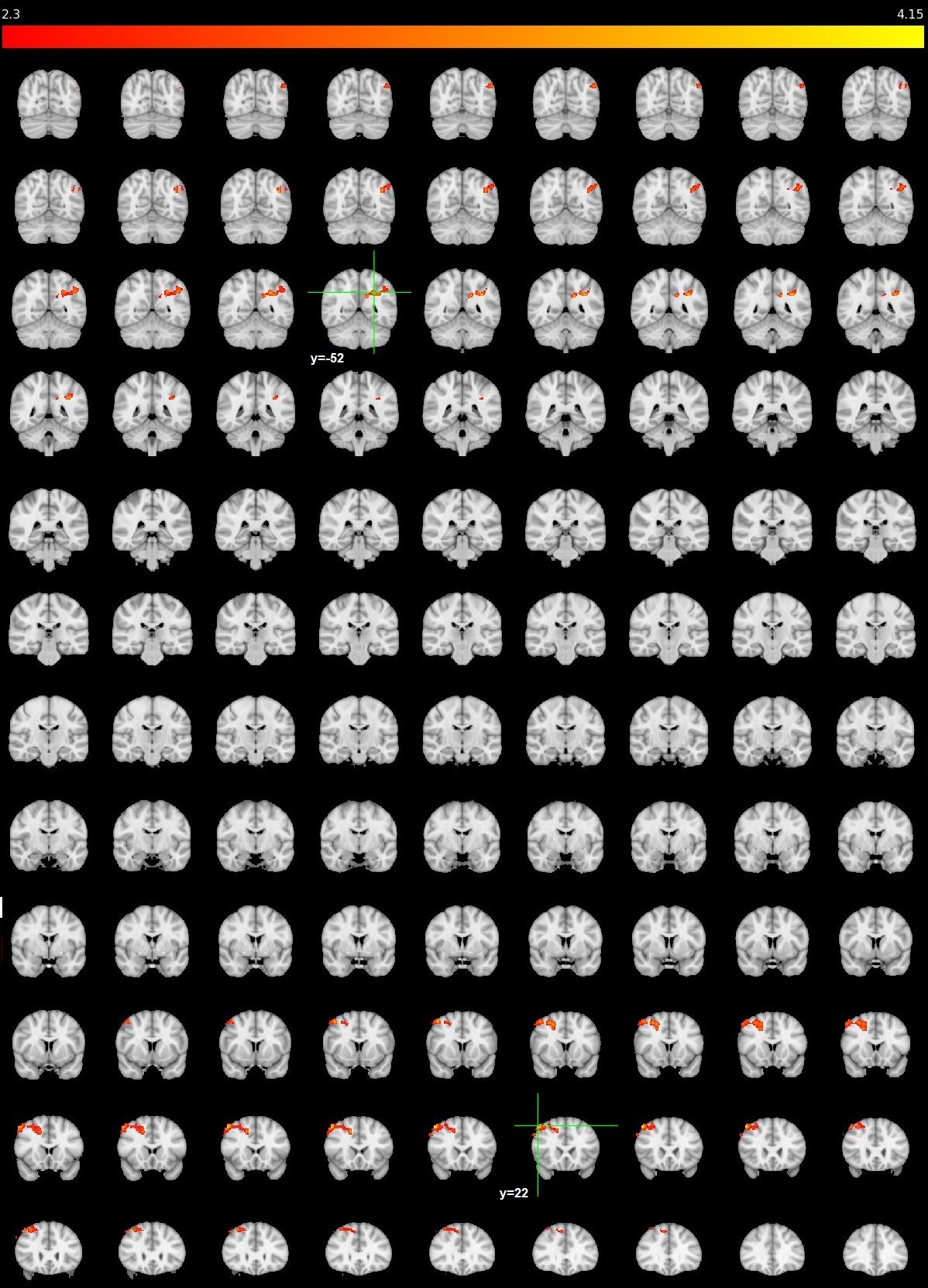


Figure SI5. Coronal views of two clusters negatively correlated with DBD symptom severity during failed inhibition versus successful go-trials, shown in radiologic view with the right brain shown on the left. The highlighted slices at y=22 and y=-52 include the voxel with maximum Z-value of 4.09 and 3.78, respectively, for these two clusters.


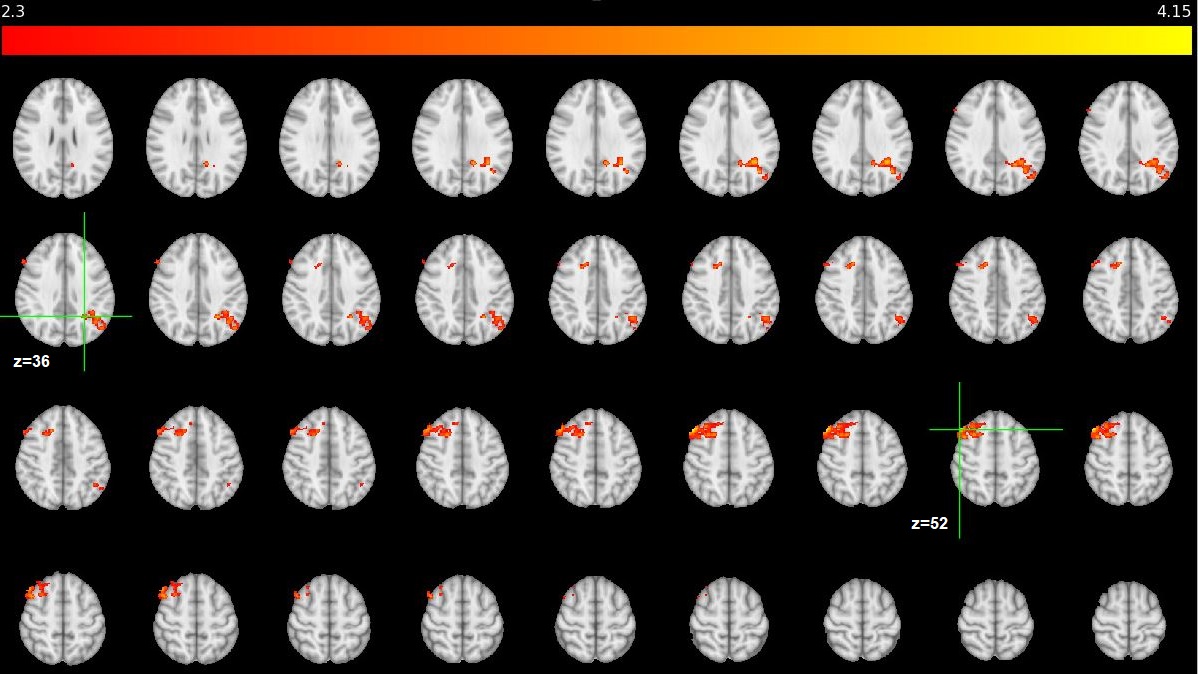


Figure SI6. Axial views of two clusters negatively correlated with DBD symptom severity during failed inhibition versus successful go-trials, shown in radiologic view with the right brain shown on the left. The highlighted slices at z=52 and z=36 include the voxel with maximum Z-value of 4.09 and 3.78, respectively, for these two clusters.


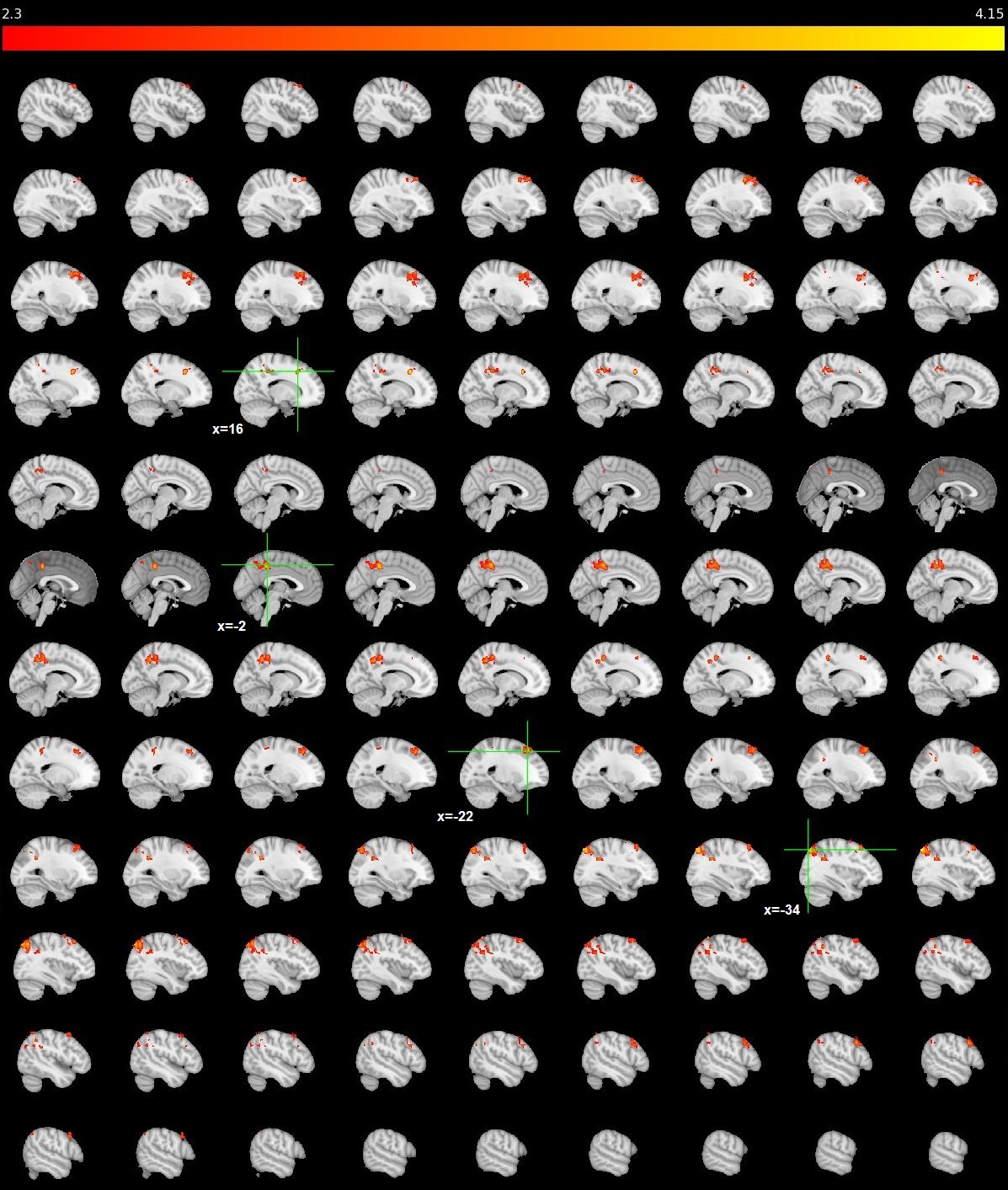


Figure SI7. Sagittal views of four clusters positively correlated with ADHD symptom severity during failed inhibition versus successful go-trials. The highlighted slices at x=-2, x=-34, x=16, and x=-22 include the voxel with maximum Z-value of 3.96, 4.15, 4.07, and 3.62, respectively, for these four clusters.


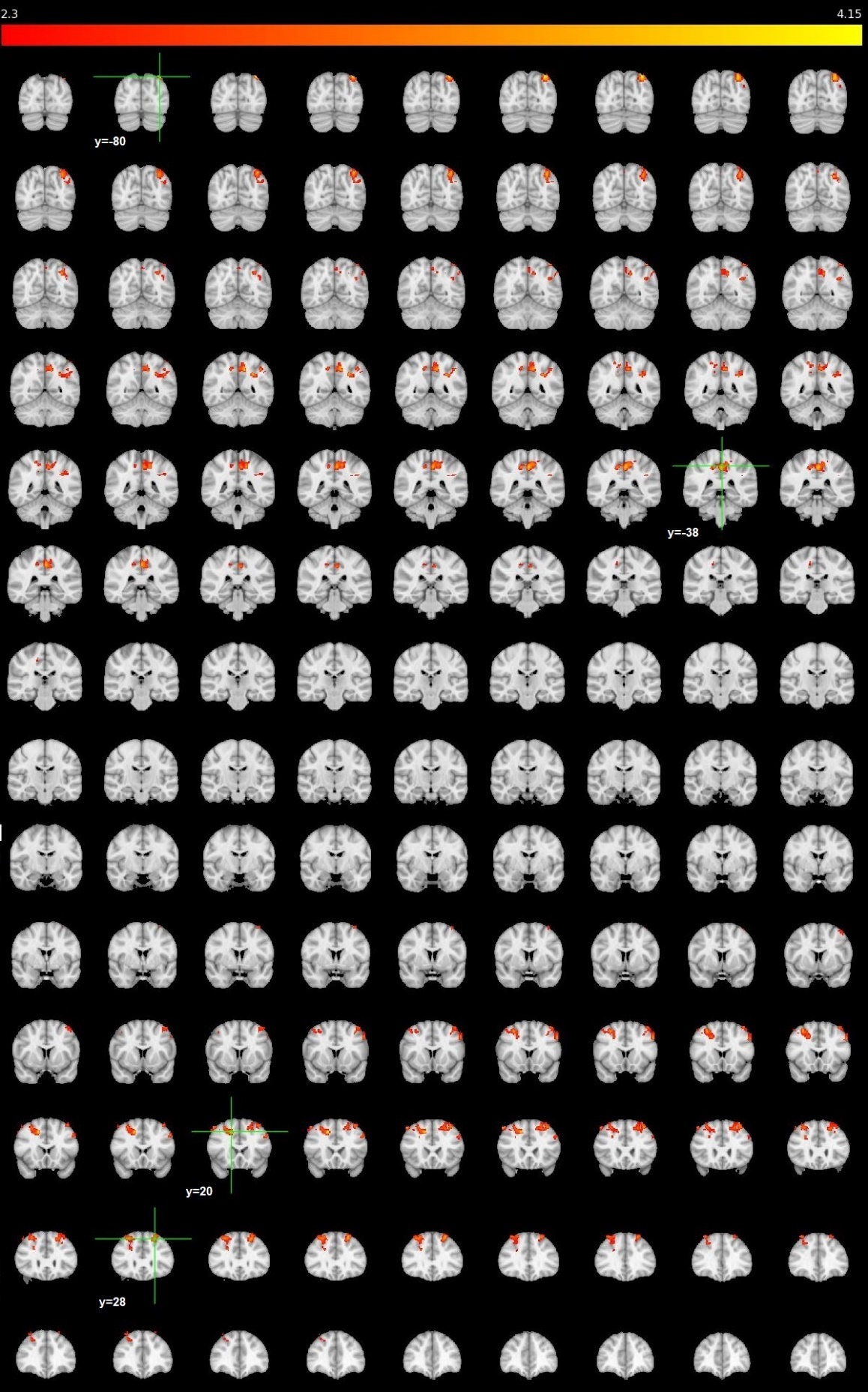


Figure SI8. Coronal views of four clusters positively correlated with ADHD symptom severity during failed inhibition versus successful go-trials, shown in radiologic view with the right brain shown on the left. The highlighted slices at y=-38, y=-80, y=20, and y=28 include the voxel with maximum Z-value of 3.96, 4.15, 4.07, and 3.62, respectively, for these four clusters.


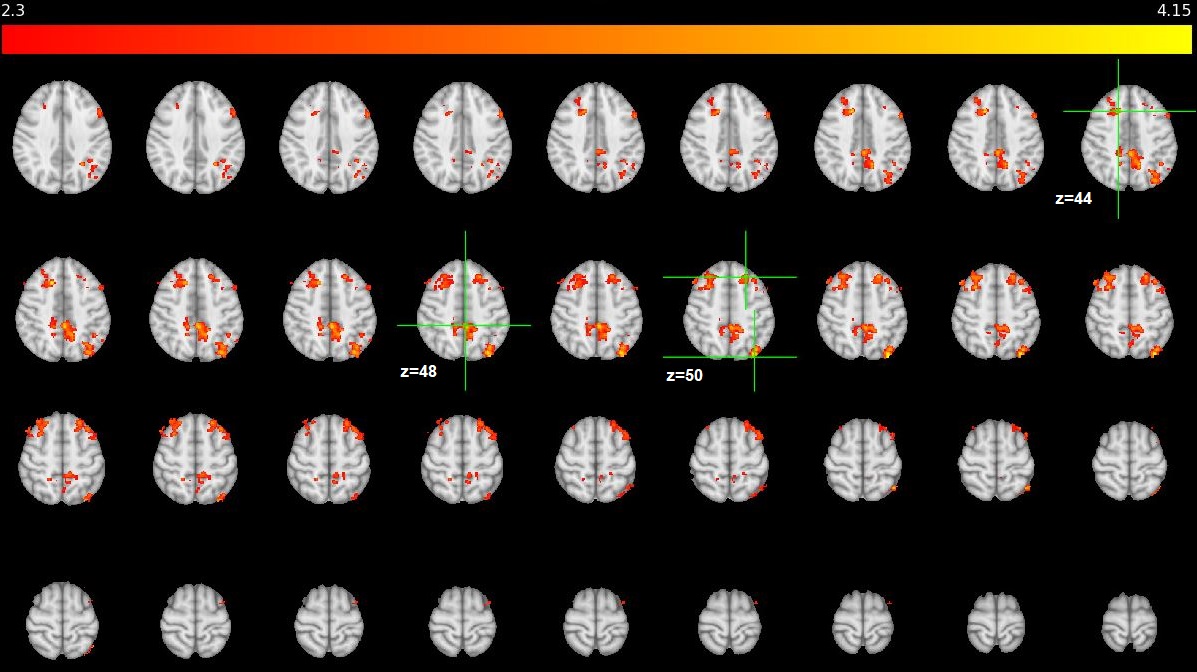


Figure SI9. Axial views of four clusters positively correlated with ADHD symptom severity during failed inhibition versus successful go-trials, shown in radiologic view with the right brain shown on the left. The highlighted slices at z=48, z=50, and z=44 include the voxel with maximum Z-value of 3.96, 4.15 (lower), 4.07, and 3.62 (upper), respectively, for these four clusters.


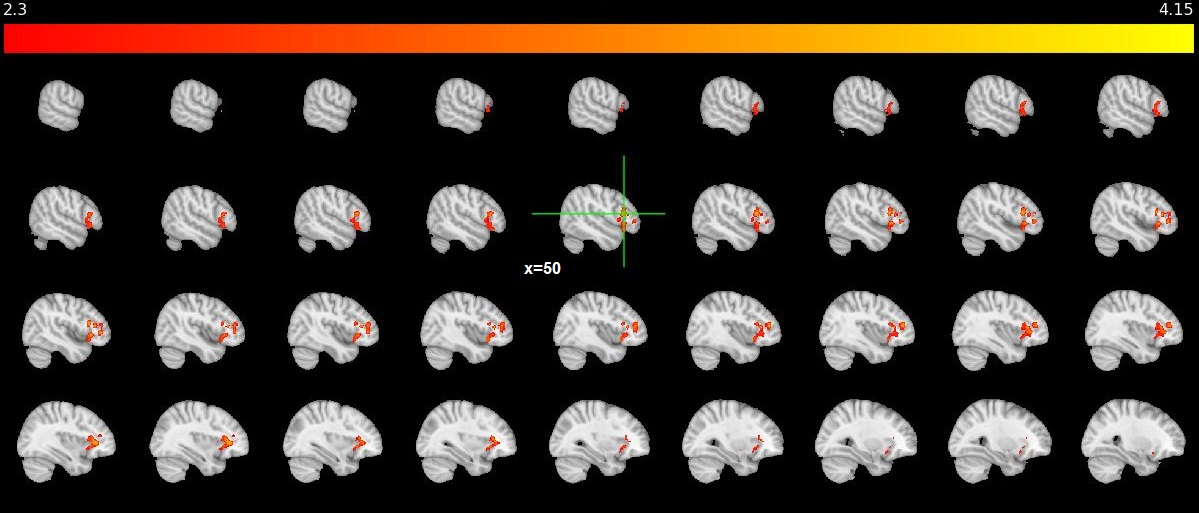


Figure SI10. Sagittal views of the cluster positively correlated with DBD symptom severity during successful inhibition versus failed inhibition. The highlighted slice at x=50 includes the voxel with maximum Z-value of 3.84 for that cluster.


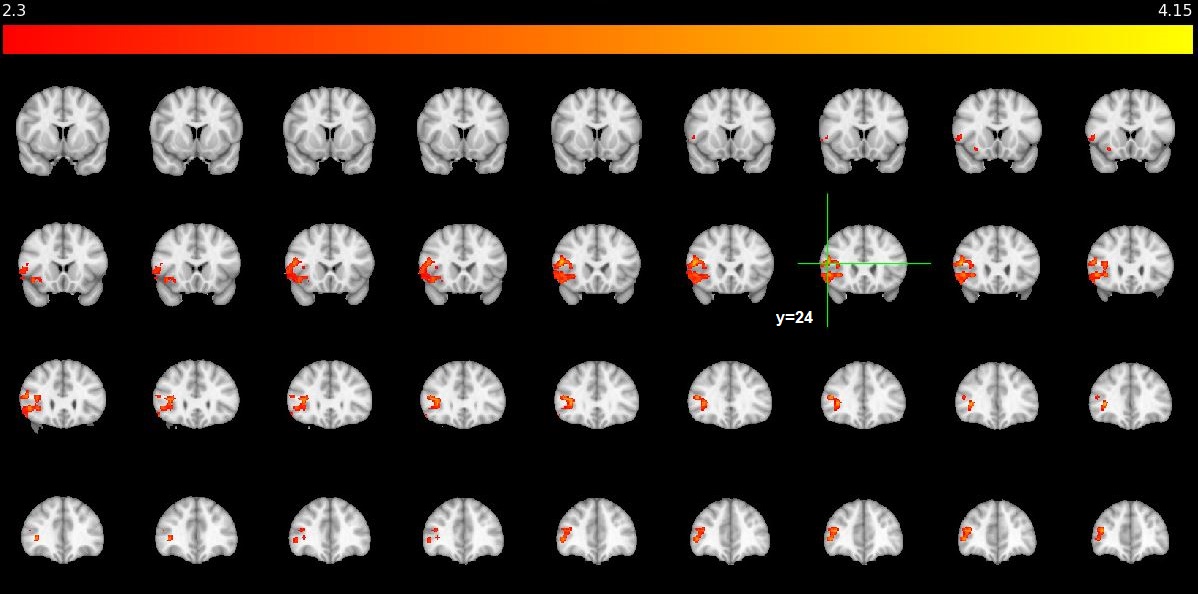


Figure SI11. Coronal views of the cluster positively correlated with DBD symptom severity during successful inhibition versus failed inhibition, shown in radiologic view with the right brain shown on the left. The highlighted slice at y=24 includes the voxel with maximum Z-value of 3.84 for that cluster.


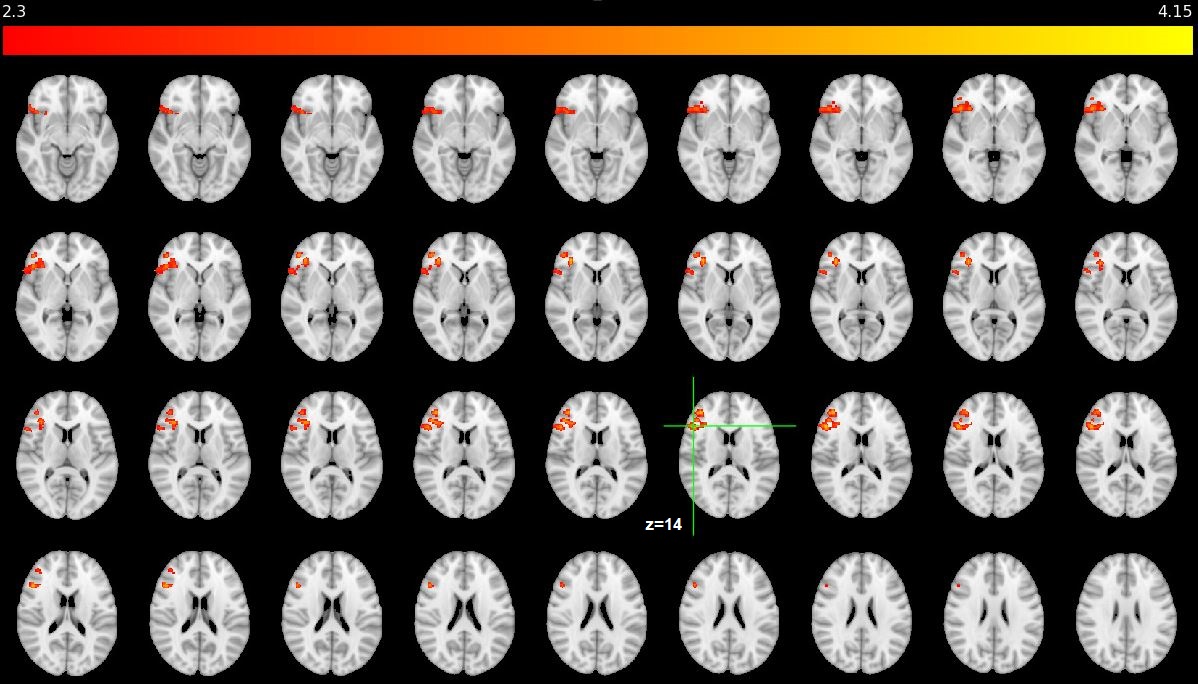


Figure SI12. Axial views of the cluster positively correlated with DBD symptom severity during successful inhibition versus failed inhibition, shown in radiologic view with the right brain shown on the left. The highlighted slice at z=14 includes the voxel with maximum Z-value of 3.84 for that cluster.


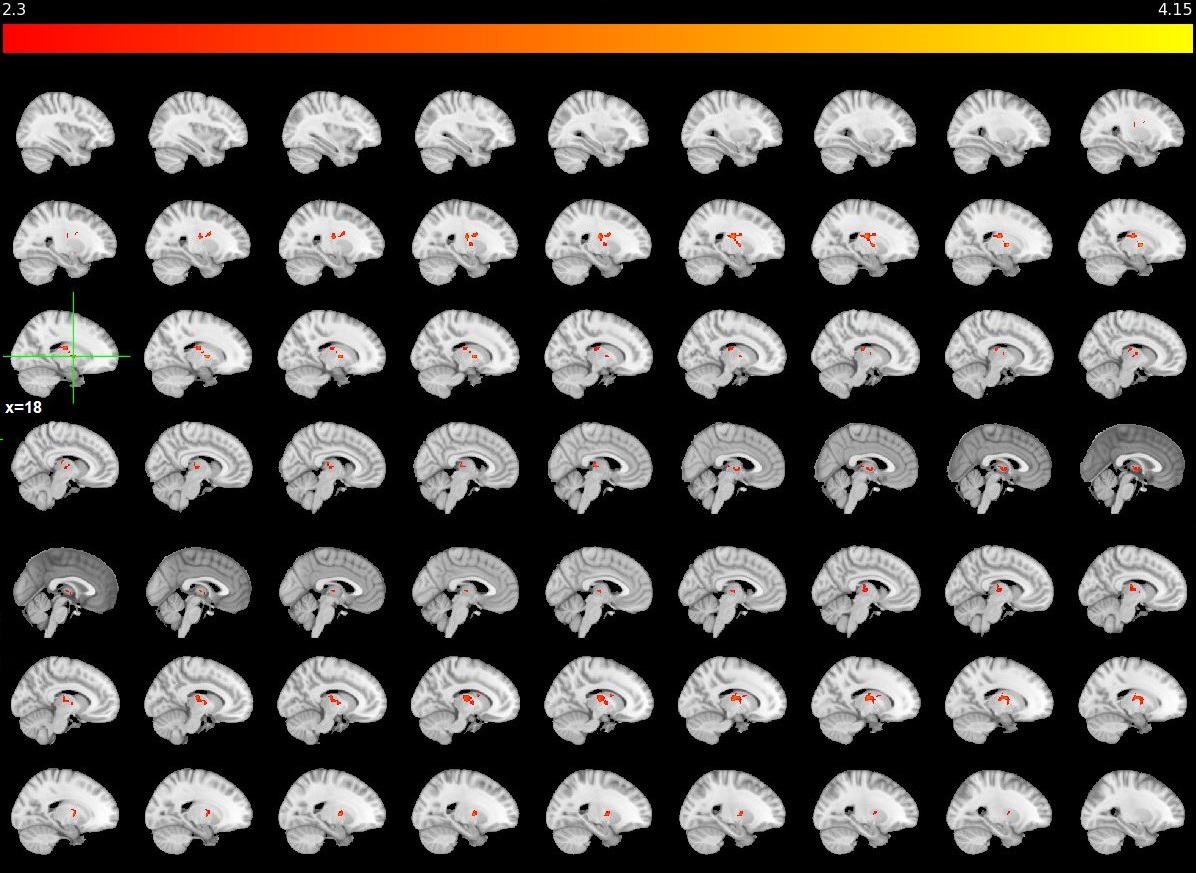


Figure SI13. Sagittal views of the cluster negatively correlated with ADHD symptom severity during successful inhibition versus failed inhibition. The highlighted slice at x=18 includes the voxel with maximum Z-value of 3.68 for that cluster.


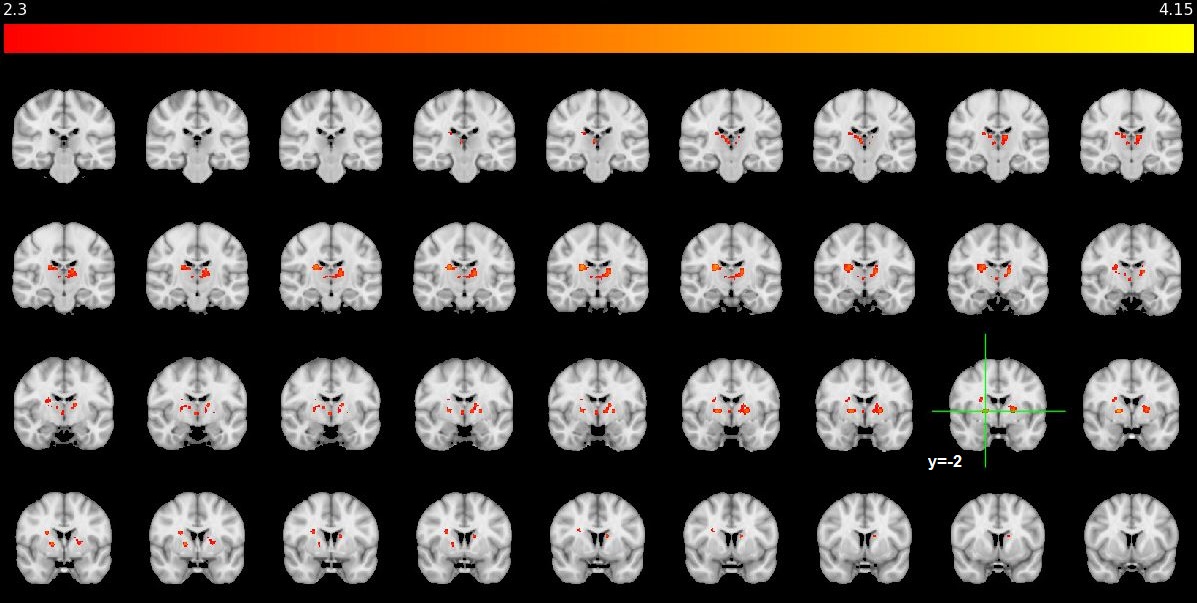


Figure SI14. Coronal views of the cluster negatively correlated with ADHD symptom severity during successful inhibition versus failed inhibition, shown in radiologic view with the right brain shown on the left. The highlighted slice at y=-2 includes the voxel with maximum Z-value of 3.68 for that cluster.


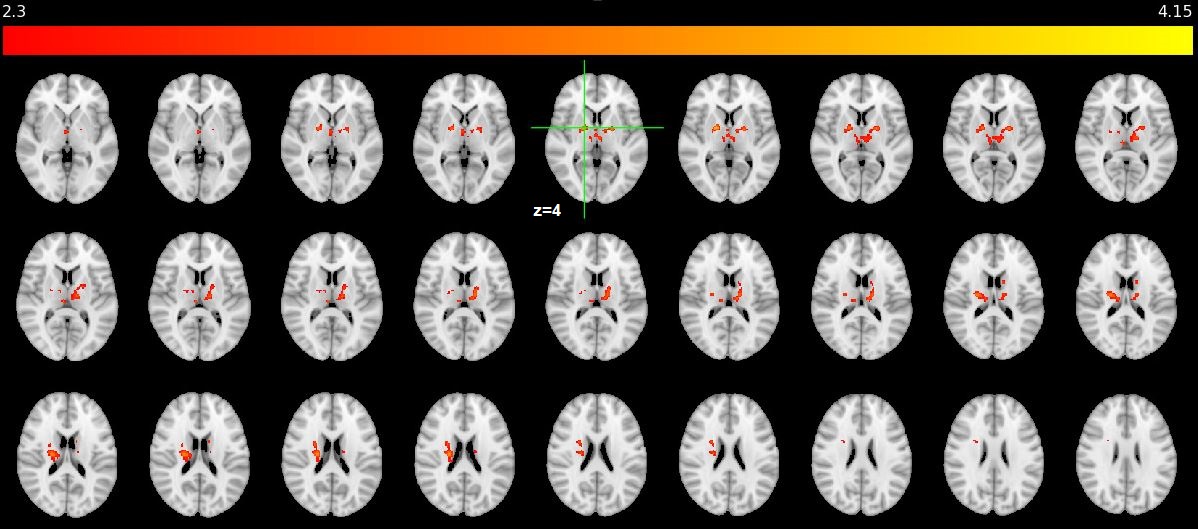


Figure SI15. Axial views of the cluster negatively correlated with ADHD symptom severity during successful inhibition versus failed inhibition, shown in radiologic view with the right brain shown on the left. The highlighted slice at z=4 includes the voxel with maximum Z-value of 3.68 for that cluster.

| Table SI1. Demographic characteristics and stop-signal task outcomes across sites | | | | | | |
| --- | --- | --- | --- | --- | --- | --- |
| Site |  | All (N=66) | Unaffected controls (N=31) | DBD (N=35) | Test statistics | p-value |
| Nijmegen | N | 25 | 14 | 11 |  |  |
|  | Sex (female/male) | 9 (36%)/16 (64%) | 6 (43%)/8 (57%) | 3 (27%)/8 (73%) | X^2^=0.649 | 0.42 |
|  | Stimulant use (yes/no) ^a^ | 5 (20%)/20 (80%) | 0/14 (100%) | 5 (45%)/6 (55%) |  |  |
|  | Handedness (right/left) | 19 (76%)/6 (24%) | 10 (71%)/4 (29%) | 9 (82%)/2 (18%) | X^2^=0.365 | 0.546 |
|  |  | (Mean [SD]) | (Mean [SD]) | (Mean [SD]) |  |  |
|  | Age in years | 14.11 [2.19] | 13.43 [2.12] | 14.98 [2.06] | *t*(23)=1.833 | 0.08 |
|  | IQ ^b^ | 102.88 [15.27] | 107.53 [15.46] | 96.98 [13.44] | *t*(23)=-1.792 | 0.086 |
|  | DBD score ^c^ | 9.94 [9.41] | 3 [4.15] | 18.76 [6.09] | *t*(23)=7.695 | <0.001 |
|  | ADHD score ^d^ | 17.74 [15.12] | 7.71 [9.08] | 30.49 [11.05] | *t*(23)=5.661 | <0.001 |
|  | MRT *(ms)* | 482.35 [118.62] | 450.12 [37.69] | 523.39 [169.16] | β=0.496 | 0.287 |
|  | ICV *(ms)* | 0.26 [0.06] | 0.25 [0.06] | 0.28 [0.05] | β=0.954 | 0.017 |
|  | Go error (%) | 3.67 [4.48] | 2.75 [3.64] | 4.84 [5.31] | β=0.228 | 0.617 |
|  | SSRT *(ms)* | 229.76 [120.92] | 223.98 [123.31] | 237.12 [123.37] | β=0.395 | 0.388 |
|  | Stop error (%) | 52.3 [5.86] | 51.25 [4.88] | 53.64 [6.92 | β=-0.052 | 0.906 |
| Mannheim | N | 16 | 6 | 10 |  |  |
|  | Sex (female/male) | 3 (19%)/13 (71%) | 1 (36%)/5 (64%) | 2 (20%)8 (80%) | X^2^=0.027 | 0.869 |
|  | Stimulant use (yes/no) ^a^ | 4 (25%)/12 (75%) | 0/6 (100%) | 4 (40%)/6 (60%) |  |  |
|  | Handedness (right/left) | 16 (100%)/0 | 6 (100%)/0 | 10 (100%)/0 |  |  |
|  |  | (Mean [SD]) | (Mean [SD]) | (Mean [SD]) |  |  |
|  | Age in years | 12.99 [2.71] | 13.84 [2.76] | 12.49 [2.69] | *t*(14)=-0.961 | 0.353 |
|  | IQ ^b^ | 108.66 [11.35] | 116.17 [8.13] | 104.15 [10.85] | *t*(14)=-2.336 | 0.035 |
|  | DBD score ^c^ | 13.06 [10.36] | 2.83 [5.23] | 19.2 [7.22] | *t*(14)=4.816 | <0.001 |
|  | ADHD score ^d^ | 18.69 [12.69] | 8 [6.99] | 25.1 [10.94] | *t*(14)=3.409 | 0.004 |
|  | MRT *(ms)* | 518.98 [104.12] | 509.69 [92.19] | 524.55 [115.12] | β=-0.543 | 0.276 |
|  | ICV *(ms)* | 0.25 [0.06] | 0.23 [0.04] | 0.25 [0.07] | β=-0.148 | 0.795 |
|  | Go error (%) | 5.45 [4.09] | 3.95 [4.12] | 6.35 [4] | β=-0.175 | 0.783 |
|  | SSRT *(ms)* | 249.05 [127.6] | 292.8 [93.91] | 222.79 [142.11] | β=-0.62 | 0.294 |
|  | Stop error (%) | 51.25 [4.83] | 50.83 [2.58] | 51.5 [5.92] | β=-0.306 | 0.649 |
| London | N | 14 | 7 | 7 |  |  |
|  | Sex (female/male) | 3 (21%)/11 (79%) | 3 (43%)/4 (57%) | 0/7 (100%) |  |  |
|  | Stimulant use (yes/no) ^a^ | 2 (14%)/12 (86%) | 0/7 (100%) | 2 (29%)/5 (71%) |  |  |
|  | Handedness (right/left) | 12 (86%)/2 (14%) | 6 (86%)/1 (14%) | 6 (86%)/1 (14%) |  |  |
|  |  | (Mean [SD]) | (Mean [SD]) | (Mean [SD]) |  |  |
|  | Age in years | 13.77 [2.44] | 13.26 [1.9] | 14.29 [2.94] | *t*(12)=0.782 | 0.45 |
|  | IQ ^b^ | 103.65 [10.47] | 107.92 [9.89] | 99.37 [9.85] | *t*(12)=-1.621 | 0.131 |
|  | DBD score ^c^ | 10.7 [8.26] | 4.86 [5.96] | 16.54 [5.74] | *t*(12)=3.736 | 0.003 |
|  | ADHD score ^d^ | 17.67 [15.29] | 7.14 [9.99] | 28.2 [12.16] | *t*(12)=3.541 | 0.004 |
|  | MRT *(ms)* | 505.53 [61.8] | 490.92 [54.11] | 520.13 [69.63] | β=0.794 | 0.219 |
|  | ICV *(ms)* | 0.27 [0.06] | 0.24 [0.06] | 0.3 [0.05] | β=0.409 | 0.513 |
|  | Go error (%) | 5.08 [4.39] | 4.76 [3.68] | 5.4 [5.28] | β=0.825 | 0.202 |
|  | SSRT *(ms)* | 231.99 [152.58] | 309.85 [97.62] | 154.13 [163.61] | β=-0.402 | 0.349 |
|  | Stop error (%) | 53.57 [9.74] | 57.14 [11.03] | 50 [7.36] | β=-0.065 | 0.859 |
| Barcelona | N | 11 | 4 | 7 |  |  |
|  | Sex (female/male) | 7 (64%)/4 (36%) | 3 (75%)/1 (25%) | 4 (57%)/3 (43%) | X^2^=0.351 | 0.554 |
|  | Stimulant use (yes/no) ^a^ | 1 (9%)/10 (91%) | 0/4 (100%) | 1 (14%)/6 (86%) |  |  |
|  | Handedness (right/left) | 11 (100%)/0 | 4 (100%)/0 | 7 (100%)/0 |  |  |
|  |  | (Mean [SD]) | (Mean [SD]) | (Mean [SD]) |  |  |
|  | Age in years | 12.49 [2.85] | 13.8 [3.14] | 11.75 [2.61] | *t*(9)=-1.667 | 0.273 |
|  | IQ ^b^ | 103.78 [9.82] | 107.63 [4.16] | 101.59 [11.69] | *t*(9)=-0.979 | 0.353 |
|  | DBD score ^c^ | 13.09 [9.12] | 3.75 [3.86] | 18.43 [6.29] | *t*(9)=4.18 | 0.002 |
|  | ADHD score ^d^ | 20.48 [11.82] | 11.7 [7.79] | 25.49 [11.04] | *t*(9)=2.183 | 0.057 |
|  | MRT *(ms)* | 527.87 [132.92] | 529 [136.57] | 527.22 [141.84] | β=0.016 | 0.973 |
|  | ICV *(ms)* | 0.33 [0.06] | 0.31 [0.06] | 0.34 [0.06] | β=0.557 | 0.354 |
|  | Go error (%) | 6.82 [5.76] | 3.37 [3.49] | 8.79 [6.06] | β=0.59 | 0.254 |
|  | SSRT *(ms)* | 160.68 [85.59] | 179.95 [53.37] | 149.68 [101.96] | β=-0.471 | 0.442 |
|  | Stop error (%) | 56.59 [8.53] | 60 [10.99] | 54.64 [6.99] | β=-0.889 | 0.098 |
| DBD, participants diagnosed with disruptive behavior disorder; DBD score, a composite DBD score; ADHD score, a composite ADHD score; MRT, mean reaction time on successful go trials; ICV, intra-individual coefficient of variation of reaction time to go stimuli; Go error, omission error percentage on go trials; SSRT, stop-signal reaction time; Stop error, error percentage on stop trials; X^2^, chi-square; t, independent-samples t-test; β, standardized regression coefficient.  ^a^ Methylphenidate and/or lisdexamphetamine use.  ^b^ Based on the Wechsler Intelligence Scale for Children III or IV [5, 6].  ^c^ Sum of the scores on the conduct and oppositional defiant problems subscales of the Child Behavior Checklist [1].  ^d^ Sum of the scores on the inattention and hyperactivity-impulsivity subscales of the Swanson, Nolan, and Pelham Rating Scale [2]. | | | | | | |

| Table SI2. T1-weighted anatomical MRI scan parameters across sites. | | | | | | |
| --- | --- | --- | --- | --- | --- | --- |
| Site | Scanner | TR/TE/TI (ms) | Flip angle | Field of view | Slices | Voxel size (mm) |
| Nijmegen | Siemens Magnetom Prisma syngo MR D13D | 2300/2.98/900 | 9 | 256 | 176 | 1x1x1.2 |
| Mannheim | Siemens Magnetom TrioTim syngo MR B17 | 2300/2.96/900 | 9 | 256 | 176 | 1x1x1.2 |
| London | GE | 2300/3.02/400 | 11 | 270 | 196 | 1x1x1.2 |
| Barcelona | Siemens Magnetom TrioTim syngo MR B17 | 2300/2.98/900 | 9 | 256 | 176 | 1x1x1.2 |

| Table SI3. T2*-weighted echo planar imaging scan parameters across sites. | | | | | | |
| --- | --- | --- | --- | --- | --- | --- |
| Site | Scanner | TR/TE (ms) | Flip angle | Field of view | Slices | Voxel size (mm) |
| Nijmegen | Siemens Magnetom Prisma syngo MR D13D | 2100/35 | 74 | 192x192 | 36 | 3x3x3 |
| Mannheim | Siemens Magnetom TrioTim syngo MR B17 | 2100/35 | 74 | 192x192 | 36 | 3x3x3 |
| London | GE | 2100/35 | 74 | 192x192 | 36 | 3x3x3 |
| Barcelona | Siemens Magnetom TrioTim syngo MR B17 | 2100/35 | 74 | 192x192 | 36 | 3x3x3 |

| Table SI4. Demographic characteristics and stop-signal task outcome measures (subjects using stimulants on the testing day were excluded) | | | | | | | | |
| --- | --- | --- | --- | --- | --- | --- | --- | --- |
|  | All (N=54) | | Unaffected controls (N=31) | | DBD (N=23) | | Test statistics | p-value |
| *Demographic characteristics* | | | | | | | | |
| Sex (female/male) | 21 (39%)/33 (61%) | | 13 (42%)/18 (58%) | | 8 (35%)/15 (65%) | | X^2^=0.284 | 0.594 |
| Handedness (right/left) | 47 (87%)/7 (13%) | | 26 (84%)/5 (16%) | | 21 (91%)/2 (9%) | | X^2^=0.647 | 0.421 |
|  | Mean | SD | Mean | SD | Mean | SD |  |  |
| Age in years | 13.38 | 2.61 | 13.52 | 2.23 | 13.2 | 3.09 | *t*(52)=-0.443 | 0.66 |
| IQ ^a^ | 105.29 | 12.55 | 109.3 | 12.15 | 99.88 | 11.16 | *t*(52)=-2.917 | 0.005 |
| DBD score ^b^ | 10 | 9.36 | 3.48 | 4.61 | 18.77 | 6.48 | *t*(52)=10.14 | <0.001 |
| ADHD score ^c^ | 15.75 | 12.98 | 8.16 | 8.48 | 25.99 | 10.8 | *t*(52)=6.801 | <0.001 |
| *Stop-signal task outcomes* | | | | | | | | |
| MRT *(ms)* | 508.89 | 104.2 | 481.04 | 73.64 | 546.44 | 127.25 | β=0.379 | 0.13 |
| ICV *(ms)* | 0.28 | 0.07 | 0.25 | 0.06 | 0.31 | 0.06 | β=816 | <0.001 |
| Go error (%) | 4.72 | 4.86 | 3.52 | 3.63 | 6.35 | 5.84 | β=0.391 | 0.124 |
| SSRT *(ms)* | 231.45 | 121.92 | 251.01 | 111.45 | 205.09 | 132.69 | β=-0.115 | 0.603 |
| Stop error (%) | 52.96 | 7.4 | 53.63 | 7.77 | 52.07 | 6.94 | β=-0.495 | 0.028 |
| DBD, participants diagnosed with disruptive behavior disorder; DBD score, a composite DBD score; ADHD score, a composite ADHD score; MRT, mean reaction time on successful go trials; ICV, intra-individual coefficient of variation of reaction time to go stimuli; Go error, omission error percentage on go trials; SSRT, stop-signal reaction time; Stop error, error percentage on stop trials; X^2^, chi-square; t, independent-samples t-test; β, standardized regression coefficient.  ^a^ Based on the Wechsler Intelligence Scale for Children III or IV [5, 6].  ^b^ Sum of the scores on the conduct and oppositional defiant problems subscales of the Child Behavior Checklist [1].  ^c^ Sum of the scores on the inattention and hyperactivity-impulsivity subscales of the Swanson, Nolan, and Pelham Rating Scale [2]. | | | | | | | | |

| Table SI5. The associations of the clusters with DBD and ADHD symptom severity (subjects using stimulants on the testing day were excluded) | | |
| --- | --- | --- |
| *Successful inhibition versus successful go trials* | | |
| Association | Brain regions | Post-hoc analyses |
| ADHD score^b^ (+) | Left superior LOC | DBD score: β=-0.437, p^a^=0.073; ADHD score: β=0.498, p^a^=0.073 |
| *Failed inhibition versus successful go trials* | | |
| Association | Brain regions | Post-hoc analyses |
| DBD score^c^ (-) | Right MFG, SFG | DBD score: β=-0.693, p^a^=0.008; ADHD score: β=0.394, p^a^=0.094 |
| DBD score^c^ (-) | Left SPL, posterior SMG, ANG, PCC | DBD score: β=-0.804, p^a^=0.001; ADHD score: β=0.61, p^a^=0.01 |
| ADHD score^b^ (+) | Bilateral PCUN | DBD score: β=-0.501, p^a^=0.051; ADHD score: β=0.535, p^a^=0.048 |
| ADHD score^b^ (+) | Left SPL, superior LOC | DBD score: β=-0.757, p^a^=0.002; ADHD score: β=0.773, p^a^=0.002 |
| ADHD score^b^ (+) | Right MFG, SFG | DBD score: β=-0.533, p^a^ =0.065; ADHD score: β=0.443, p^a^=0.048 |
| ADHD score^b^ (+) | Left SFG, MFG | DBD score: β=-0.485, p^a^=0.037; ADHD score: β=0.603, p^a^=0.022 |
| *Successful inhibition versus failed inhibition* | | |
| Association | Brain regions | Post-hoc analyses |
| DBD score^c^ (+) | Right IFGtri, FPo, FO, anterior INS | DBD score: β=0.739, p^a^=0.004; ADHD score: β=-0.245, p^a^=0.281 |
| ADHD score^b^ (-) | Bilateral THA | DBD score: β=0.833, p^a^<0.001; ADHD score: β=-1.053, p^a^<0.001 |
| MNI, Montreal Neurological Institute; DBD score, a composite DBD score; ADHD score, a composite ADHD score; (+), positive association; (-), negative association; β, standardized regression coefficient.  Brain regions: ANG, angular gyrus; FO, frontal operculum; FPo, frontal pole; IFGtri, inferior frontal gyrus, triangular part; INS, insula; LOC, lateral occipital cortex; MFG, middle frontal gyrus; PCC, posterior cingulate gyrus; PCUN, precuneus; SFG, superior frontal gyrus; SMG, supramarginal gyrus; SPL, superior parietal lobule; THA, thalamus.  ^a^ FDR corrected p-value [7].  ^b^ Sum of the scores on the inattention and hyperactivity-impulsivity subscales of the Swanson, Nolan, and Pelham Rating Scale [2].  ^c^ Sum of the scores on the conduct and oppositional defiant problems subscales of the Child Behavior Checklist [1]. | | |

| Table SI6. The associations of the clusters with DBD and ADHD symptom severity, controlling for aggression and callous-unemotional traits | | | |
| --- | --- | --- | --- |
| *Successful inhibition versus successful go trials* | | | |
| Association | Brain regions | Controlling for aggression^a^ | Controlling for callous-unemotional traits^b^ |
| ADHD score^c^ (+) | Left superior LOC | DBD score: β=-0.402, p^e^=0.061  ADHD score: β=0.607, p^e^=0.01 | DBD score: β=-0.446, p^e^=0.04  ADHD score: β=0.572, p^e^=0.018 |
| *Failed inhibition versus successful go trials* | | | |
| Association | Brain regions | Controlling for aggression^a^ | Controlling for callous-unemotional traits^b^ |
| DBD score^d^ (-) | Right MFG, SFG | DBD score: β=-0.848, p^e^<0.001  ADHD score: β=0.575, p^e^=0.004 | DBD score: β=-0.864, p^e^<0.001  ADHD score: β=0.555, p^e^=0.006 |
| DBD score^d^ (-) | Left SPL, posterior SMG, ANG, PCC | DBD score: β=-0.785, p^e^<0.001  ADHD score: β=0.657, p^e^=0.002 | DBD score: β=-0.786, p^e^<0.001  ADHD score: β=0.679, p^e^=0.001 |
| ADHD score^c^ (+) | Bilateral PCUN | DBD score: β=-0.552, p^e^=0.01  ADHD score: β=0.744, p^e^ 0.001 | DBD score: β=-0.627, p^e^=0.005  ADHD score: β=0.688, p^e^=0.004 |
| ADHD score^c^ (+) | Left SPL, superior LOC | DBD score: β=-0.665, p^e^<0.001  ADHD score: β=0.879, p^e^<0.001 | DBD score: β=-0.72, p^e^<0.001  ADHD score: β=0.861, p^e^<0.001 |
| ADHD score^c^ (+) | Right MFG, SFG | DBD score: β=-0.566, p^e^=0.009  ADHD score: β=0.568, p^e^=0.009 | DBD score: β=-0.58, p^e^=0.01  ADHD score: β=0.551, p^e^=0.01 |
| ADHD score^c^ (+) | Left SFG, MFG | DBD score: β=-0.473, p^e^=0.017  ADHD score: β=0.712, p^e^<0.001 | DBD score: β=-0.465, p^e^=0.018  ADHD score: β=0.734, p^e^<0.001 |
| *Successful inhibition versus failed inhibition* | | | |
| Association | Brain regions | Controlling for aggression^a^ | Controlling for callous-unemotional traits^b^ |
| DBD score^d^ (+) | Right IFGtri, FPo, FO, anterior INS | DBD score: β=0.744, p^e^<0.001  ADHD score: β=-0.242, p^e^=0.209 | DBD score: β=0.722, p^e^<0.001  ADHD score: β=-0.27, p^e^=0.164 |
| ADHD score^c^ (-) | Bilateral THA | DBD score: β=0.582, p^a^=0.006  ADHD score: β=-0.819, p^e^<0.001 | DBD score: β=0.508, p^e^=0.013  ADHD score: β=-0.885, p^e^<0.001 |
| MNI, Montreal Neurological Institute; DBD score, a composite DBD score; ADHD score, a composite ADHD score; (+), positive association; (-), negative association; β, standardized regression coefficient.  Brain regions: ANG, angular gyrus; FO, frontal operculum; FPo, frontal pole; IFGtri, inferior frontal gyrus, triangular part; INS, insula; LOC, lateral occipital cortex; MFG, middle frontal gyrus; PCC, posterior cingulate gyrus; PCUN, precuneus; SFG, superior frontal gyrus; SMG, supramarginal gyrus; SPL, superior parietal lobule; THA, thalamus.  ^a^ Total score on the Reactive-Proactive Aggression Questionnaire [3].  ^b^ Total score on the Inventory of Callous-Unemotional Traits [4].  ^c^ Sum of the scores on the inattention and hyperactivity-impulsivity subscales of the Swanson, Nolan, and Pelham Rating Scale [2].  ^d^ Sum of the scores on the conduct and oppositional defiant problems subscales of the Child Behavior Checklist [1].  ^e^ FDR corrected p-value [7]. | | | |

| Table SI7. Demographic characteristics and stop-signal task outcome measures [comparison between included and excluded participants due to excessive head motion (N=30) and extreme scores on behavioral performance (N=7)] | | | | | | |
| --- | --- | --- | --- | --- | --- | --- |
|  | Included participants (N=66) | | Excluded participants (N=37) | | Test statistics |  |
| *Demographic characteristics* | | | | | | |
| Sex (female/male) | 22 (33%)/44 (67%) | | 13 (35%)/24 (65%) | | X^2^=0.034 |  |
| Handedness (right/left) | 58 (88%)/8 (12%) | | 32 (87%)/5 (13%) | | X^2^=0.042 |  |
|  | Mean | SD | Mean | SD |  |  |
| Age in years | 13.5 | 2.51 | 12.53 | 2.92 | *t*(101)=-1.773 |  |
| IQ ^a^ | 104.59 | 12.57 | 103.33 | 11.58 | *t*(101)=-0.502 |  |
| DBD score ^b^ | 11.38 | 9.27 | 14.62 | 10.18 | *t*(101)=1.643 |  |
| ADHD score ^c^ | 18.41 | 13.82 | 22.64 | 17.43 | *t*(101)=1.354 |  |
| *Stop-signal task outcomes* | | | | | | |
| MRT *(ms)* | 503.7 | 107.29 | 563.98 | 170.77 | *t*(101)=2.2 |  |
| ICV *(ms)* | 0.27 | 0.06 | 0.325 | 0.091 | *t*(101)=3.69 |  |
| Go error (%) | 5.92 | 4.64 | 8.24 | 7.53 | *t*(101)=1.936 |  |
| SSRT *(ms)* | 223.4 | 125.69 | 166.74 | 265.3 | *t*(101)=-1.469 |  |
| Stop error (%) | 53.03 | 7.17 | 52.36 | 16.49 | *t*(101)=-0.286 |  |
| DBD, participants diagnosed with disruptive behavior disorder; DBD score, a composite DBD score; ADHD score, a composite ADHD score; MRT, mean reaction time on successful go trials; ICV, intra-individual coefficient of variation of reaction time to go stimuli; Go error, omission error percentage on go trials; SSRT, stop-signal reaction time; Stop error, error percentage on stop trials; X^2^, chi-square; t, independent-samples t-test; β, standardized regression coefficient.  ^a^ Based on the Wechsler Intelligence Scale for Children III or IV [5, 6].  ^b^ Sum of the scores on the conduct and oppositional defiant problems subscales of the Child Behavior Checklist [1].  ^c^ Sum of the scores on the inattention and hyperactivity-impulsivity subscales of the Swanson, Nolan, and Pelham Rating Scale [2]. | | | | | | |

**References**

1. Achenbach TM, Rescorla LA. *Manual for the ASEBA school-age forms & profiles: an integrated system of multi-informant assessment*. Burlington, VT: University of Vermont Research Center for Children, Youth, & Families; 2001. ISBN: 0938565737
2. Swanson JM, Schuck S, Porter MM, Carlson C, Hartman CA, Sergeant JA, et al. Categorical and dimensional definitions and evaluations of symptoms of ADHD: history of the SNAP and the SWAN rating scales. *The International journal of educational and psychological assessment*. 2012;10(1):51-70.
3. Raine A, Dodge K, Loeber R, Gatzke‐Kopp L, Lynam D, Reynolds C, Stouthamer‐Loeber, M, Liu, J. The reactive–proactive aggression questionnaire: Differential correlates of reactive and proactive aggression in adolescent boys. *Aggressive Behavior: Official Journal of the International Society for Research on Aggression*. 2006;*32*(2):159-171. doi:10.1002/ab.20115
4. Essau CA, Sasagawa S, Frick PJ. Callous-unemotional traits in a community sample of adolescents. *Assessment.* 2006;13(4):454-469. doi:10.1177/1073191106287354
5. Wechlser D. *Manual for the Wechsler Intelligence Scale for Children-Third Edition, (WISC-III).* San Antonio, TX: The Psychological Corporation; 1991.
6. Wechsler D. *Wechsler intelligence scale for children – Fourth edition (WISC-IV).* San Antonio, TX: The Psychological Corporation; 2003.
7. Benjamini Y, Hochberg Y. Controlling the false discovery rate: a practical and powerful approach to multiple testing. *Journal of the Royal statistical society: series B (Methodological)*. 1995;57(1):289-300. doi:10.1111/j.2517-6161.1995.tb02031.x
